# Pathogen local adaptation shapes Pierce’s disease of grapevines outcomes under field conditions

**DOI:** 10.1128/aem.01520-26

**Published:** 2026-09-16

**Authors:** Monica A. Donegan, Alexandra K. Kahn, Andrea L. M. Brown, Mamata KC, Leonardo De La Fuente, Monica L. Cooper, Matthew P. Daugherty, Rodrigo P. P. Almeida

**Affiliations:** 1 Department of Environmental Science, Policy, and Management, University of California-Berkeley 1438, Berkeley, California, USA; 2 Department of Entomology and Plant Pathology, Auburn University 606209, Auburn, Alabama, USA; 3 Division of Agriculture and Natural Resources, University of California Cooperative Extension, Napa, California, USA; 4 Department of Entomology, University of California-Riverside 8790 , Riverside, California, USA; The University of Arizona, Tucson, Arizona, USA

**Keywords:** bacterial plant pathogen, local adaptation, plant disease, thermal adaptation, twitching motility, Type IV pili, *Xylella fastidiosa*

## Abstract

Climate change is broadly expected to increase the range of many plant diseases, yet the current status of local thermal adaptation in many pathogens is poorly understood. *Xylella fastidiosa* (*Xf*) is a global bacterial plant pathogen that causes Pierce’s disease (PD) of grapevines and infects over 700 other host plant species, impacting both agricultural and natural ecosystems. In a common garden experiment with 477 vines in the field, we compared PD outcomes from a local (colder climate in CA) vs non-local (warmer climate in CA) bacterial strain in 13 Mediterranean grapevine varieties over 3 years. Relative to the local strain, there was 77% lower overwinter survival in the non-local strain from a warmer climate, strongly indicating local adaptation in these CA *Xf* populations. Host genotype also had a significant effect on pathogen winter survival, and grapevine varieties differed in PD susceptibility. Additionally, we assessed *in planta* evolution of the two pathogen strains over 3 years by whole-genome sequencing 58 field-derived isolates. There were convergent loss-of-function mutations in genes encoding minor Type IV pilin (T4P) proteins, which control twitching motility and other virulence phenotypes, suggesting rapid adaptive evolution. Our results suggest local adaptation to cold temperatures in a bacterial plant pathogen and a possible role for minor Type IV pilins in thermal adaptation. These findings demonstrate the urgent need to incorporate *X. fastidiosa* evolution and local thermal adaptation into global models of PD spread. Differentiating pathotypes with distinct thermal adaptations will improve disease forecasting and inform quarantine decisions.

**IMPORTANCE:**

Forecasting the movement of plant pathogens is a critical issue under global warming to effectively manage future plant disease outbreaks. Yet, current plant pathogen local thermal adaptation is often unaccounted for, especially in bacterial pathogens. Our study examines local adaptation to temperature in a bacterial plant pathogen, *Xylella fastidiosa,* that causes disease in grapevines in addition to infecting 700 other plant species. In a large-scale field experiment across 13 grapevine varieties, we demonstrate local adaptation in pathogen winter survival in distinct *Xylella fastidiosa* strains. Additionally, we found evidence of adaptive evolution in just 3 years, as we observed convergent mutations after resequencing strains that evolved in the field. Our results suggest that *X. fastidiosa* populations—even within a small geographic area—have distinct adaptations to winter temperatures and may exhibit differential responses to warming winters.

## INTRODUCTION

Climate change is broadly expected to increase global plant disease pressure (1, 2). Yet, the impact of current local adaptation to temperature under a changing climate has been overlooked in bacterial plant pathogens. Even within a small geographic region, pathogen populations may be best adapted to local thermal conditions such as winter temperatures. Despite vast implications for management of plant pathogens, local thermal adaptation in nature is understudied in microbial systems, especially in bacterial species (3).

For the bacterial plant pathogen *Xylella fastidiosa* (*Xf*), temperature strongly influences fitness (4), possibly resulting in populations that are adapted to local temperatures (5). *Xf* causes Pierce’s disease (PD) of grapevines (*Vitis vinifera*) and infects over 700 species of host plants in both agricultural and natural systems (6). Vines can succumb to this lethal disease within a few years, as sap movement is impaired by blocked xylem vessels, which result from both plant immune responses and bacterial aggregates (7). PD pressure is higher in warmer viticultural areas because *Xf* growth and symptom development are temperature-dependent (4, 8). On the other hand, in colder viticultural areas, infected grapevines exposed to sufficiently cold winters can fully recover from PD (9, 10). This phenomenon, known as overwinter recovery, involves the grapevine recovering from *Xf* infection; that is, overwinter recovery in the vine is the inverse of bacterial winter survival. In colder climates, there may be selection for *Xf* populations to adapt to better survive cold winters. While the exact duration of exposure and cold temperatures for pathogen death to occur are unclear (11), the influence of temperature on *Xf* winter survival has long been established (4, 9–13).

Host genotypes can also influence PD susceptibility (both symptom severity and disease incidence) within the growing season, overwinter *Xf* survival, and/or both traits. Grapevine varieties differ in susceptibility to PD (14–16). Slight differences in overwinter *Xf* survival were also observed between some varieties (11, 17–19), but few varieties have been tested.

The influence of pathogen genotypes on PD outcomes is more nuanced. Based on genomic and experimental evidence, populations of *Xf* in California seem to be locally adapted to temperature (5, 19). Initially, under a hot climate in the Central Valley of California, a field study comparing several geographically distinct *Xf* populations did not show differences in virulence or pathogen winter survival (20). However, in potted grapevines comparing a novel *Xf* population from a colder region (Mendocino County) to a population from the Central Valley (Kern County), there was evidence of local adaptation and differences in virulence (19). In addition to pathogen genotype, another relevant pathogen factor is bacterial load, which is influenced by host susceptibility (21). A larger bacterial pathogen load before the winter has been hypothesized to increase *Xf* winter survival (13, 17, 21) but has never been shown empirically.

**Fig 1:**
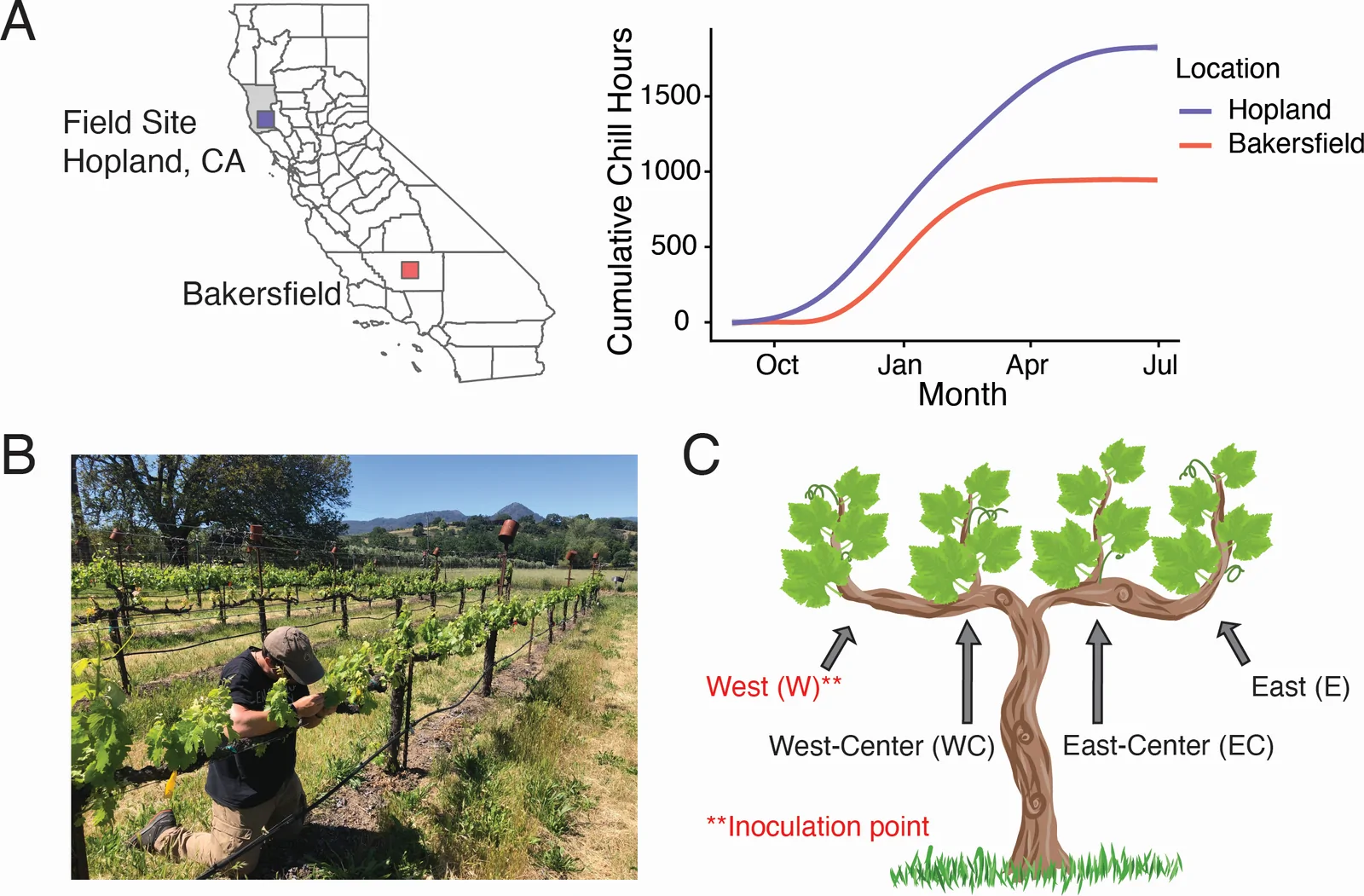
Experimental location and setup. (**A**) The experiment vineyard (gray: Mendocino County) was in Hopland, CA, the location where the local strain (D06) was isolated. The non-local strain (Je115) was isolated from Bakersfield, CA. Temperature data from data loggers averaged over the 1991–2026 winters in both locations show cumulative chill hours (below 7.2°C/45°F). (**B**) Vines during mechanical (needle) inoculation on 4 May 2021. (**C**) Vines were inoculated on the Western-most shoot, then sampled and evaluated in all quadrants.

To further evaluate local adaptation to climate in *X. fastidiosa*, we performed a common garden experiment (22, 23) at a vineyard with cold winters at the Northern range edge of PD in California (Fig. 1A). In this experiment, we compared a local and non-local strain across 13 Mediterranean grapevine varieties to untangle the effects of host and pathogen genotypes on PD susceptibility and pathogen overwinter survival. The two *Xf* strains are genetically distinct (24), and the non-local strain was isolated from a warmer region in California. In potted grapevines, both genotypes were previously tested in a reciprocal transplant experiment in their respective local climates, revealing differences in winter survival consistent with local adaptation but mediated by two host genotypes (19). We built on those results by testing in mature fields in the field, as well as across 13 grapevine varieties. In a colder viticulture region at our field site, we predicted that the potentially cold-adapted local strain would survive the cold winters at greater rates than the non-local strain. Beyond possible local adaptation, we leveraged the field setup to measure bacterial evolution during host infection under natural conditions by sequencing field-evolved isolates to assess in-field mutation rate and short-term evolution. Our goal was to examine *Xf* local adaptation to temperature across distinct strains and possible adaptive evolution in the field. Understanding both current local adaptation and adaptive potential in this pathogen is critical to managing *Xf* diseases under climate change.

## RESULTS

### Disease susceptibility and progression differ across varieties

**Fig 2:**
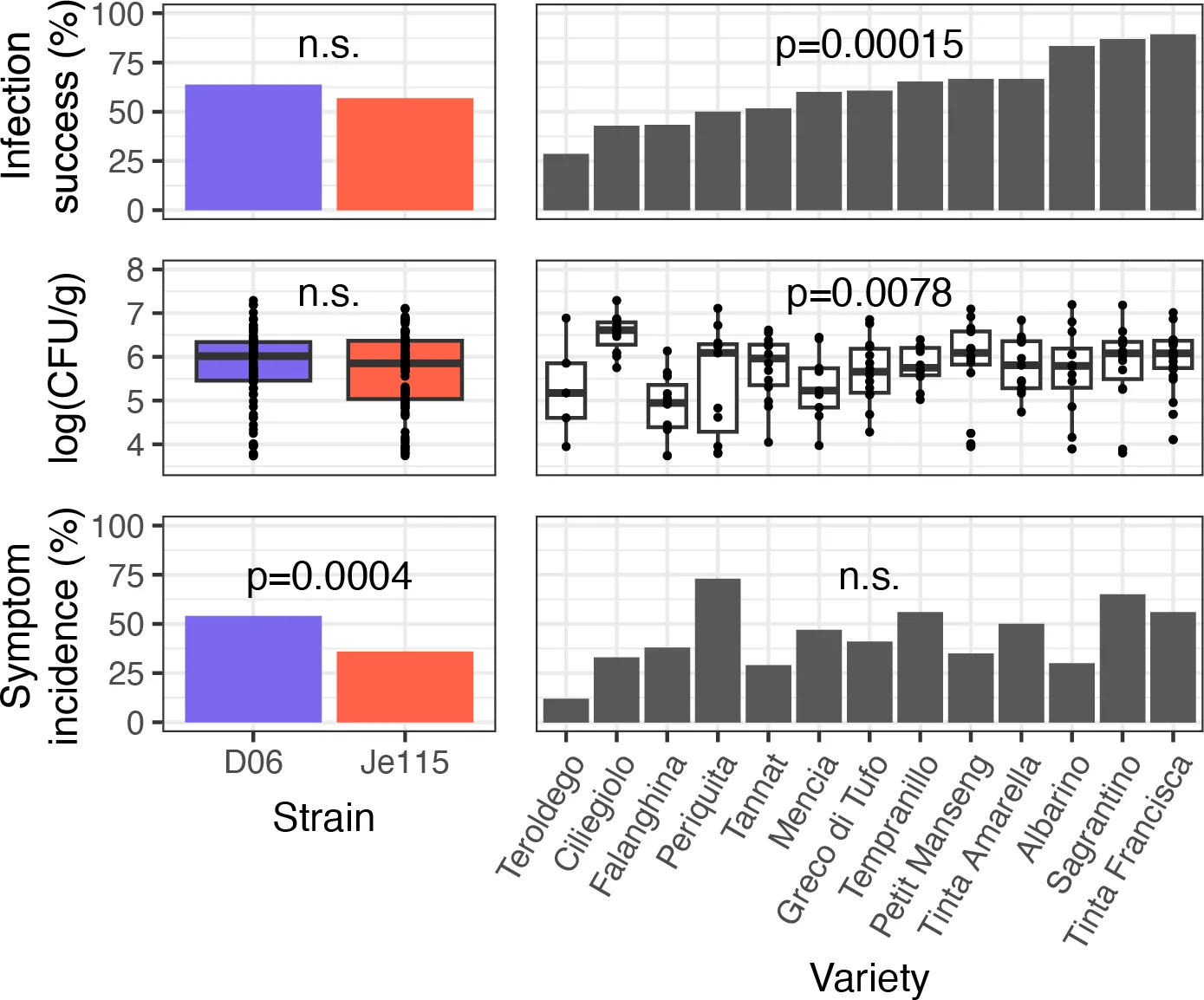
Susceptibility to PD by variety and pathogen genotype in the first year. All metrics are from the first year (2021), prior to winter exposure. From top to bottom, infection success (no. of positive vines/no. of vines inoculated), bacterial population size (log CFU/g) in positive vines, and symptom incidence (no. of symptomatic/no. of positive vines) among positive vines split by (left) *Xf* strain and (right) grapevine variety. Statistical values for infection success and symptom incidence are the results of Wald’s Chi-square test from generalized linear mixed models; statistical values for bacterial population sizes are from deviance Chi-square tests in a linear mixed model.

We compared disease progression of local (D06) and non-local (Je115) *Xf* strains at an experimental vineyard in Hopland, CA (Fig. 1A), using vines (*n* = 477) from 13 European grapevine varieties (Fig. S1). The non-local strain was from Bakersfield, CA, where winters are warmer than at the field experiment location (Fig. 1A). In the first year, infection success did not vary significantly between the two strains (D06, 111/174; Je115, 99/174) but was significantly different across the 13 varieties (𝛸^2^ = 38.07, df = 12, *P* < 0.0001) (Fig. 2). Similarly, bacterial population sizes among positive vines differed by variety (*F*_12, 150_= 2.379, *P* = 0.0078) but not by strain (Fig. 2). By contrast, in the first year, symptom incidence in positive vines was greater (𝛸^2^ = 12.617, df = 1, *P* = 0.0004) in vines infected with the local D06 strain (60%: 67/111) compared to the non-local Je115 strain (39%; 39/99), while variety was not significant (Fig. 2). These three metrics of susceptibility (infection success, bacterial population sizes, and symptom incidence in positive plants) were all positively correlated, but not significantly correlated (Fig. S9).

**Fig 3:**
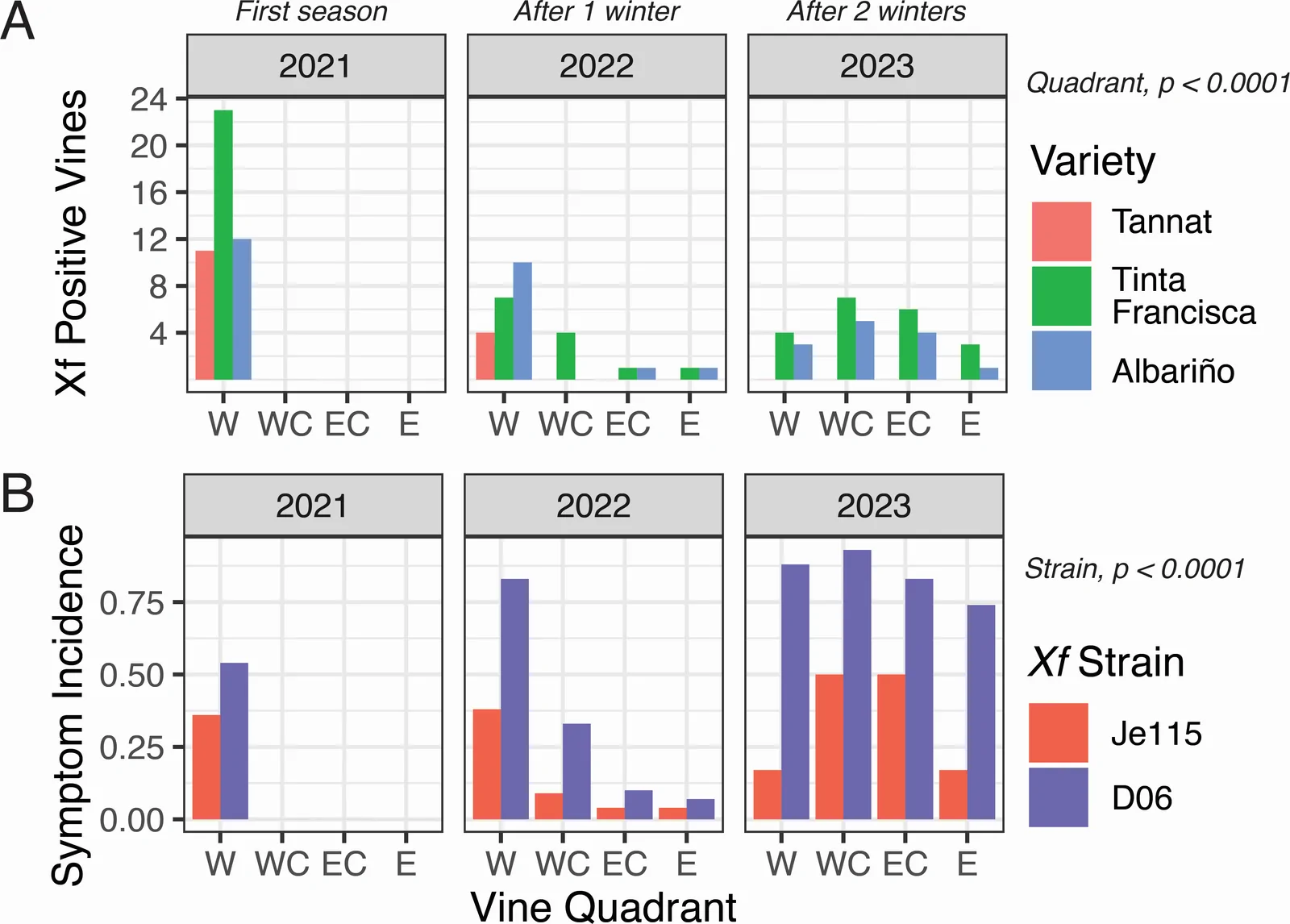
Pierce’s disease progression over 3 years. (**A**) Infection status of vine quadrants of three varieties, determined by qPCR. (**B**) Symptom incidence in *Xf*-positive vines (in all 13 varieties) by strain. Incidence proportions are only among vines that were positive in that year. The *P*-values on the right of panels A and B are the results of Wald’s Chi-square tests from GLMM models using all 3 years of data.

Symptoms and cross-vine movement progressed throughout the 3 years of monitoring after inoculation in the Westernmost shoot (Fig. 1B and C). We observed classical PD symptoms such as marginal leaf scorch and shriveled berries, recently recognized symptoms like early-season stunting (17), and non-canonical symptoms such as a barren cane (Fig. S10 to S12). In both April and May of 2022 and 2023, the normalized shoot height in the Western quadrant was significantly shorter (by ~17% in 2022 and by ~40% in 2023) in infected compared to uninfected vines (𝛸^2^ = 13.1615, df = 1, *P* = 0.0003), demonstrating early-season stunting. Both pathogen detection and symptoms were restricted to the quadrant of inoculation (W) in the first year, and limited spread was detected in the second year (Fig. 3A and B). However, by the third year, both strains were detected throughout the vine, as were symptoms (Fig. 3A and B). Across all years, greater symptom incidence was observed in vines infected with the local D06 strain than with the Je115 strain (𝛸^2^ = 57.59, df = 1, *P* < 0.0001), particularly after winter exposure in 2022 and 2023 (Fig. 3B).

### Strong effects of host and pathogen genotypes on pathogen overwinter survival

**Fig 4:**
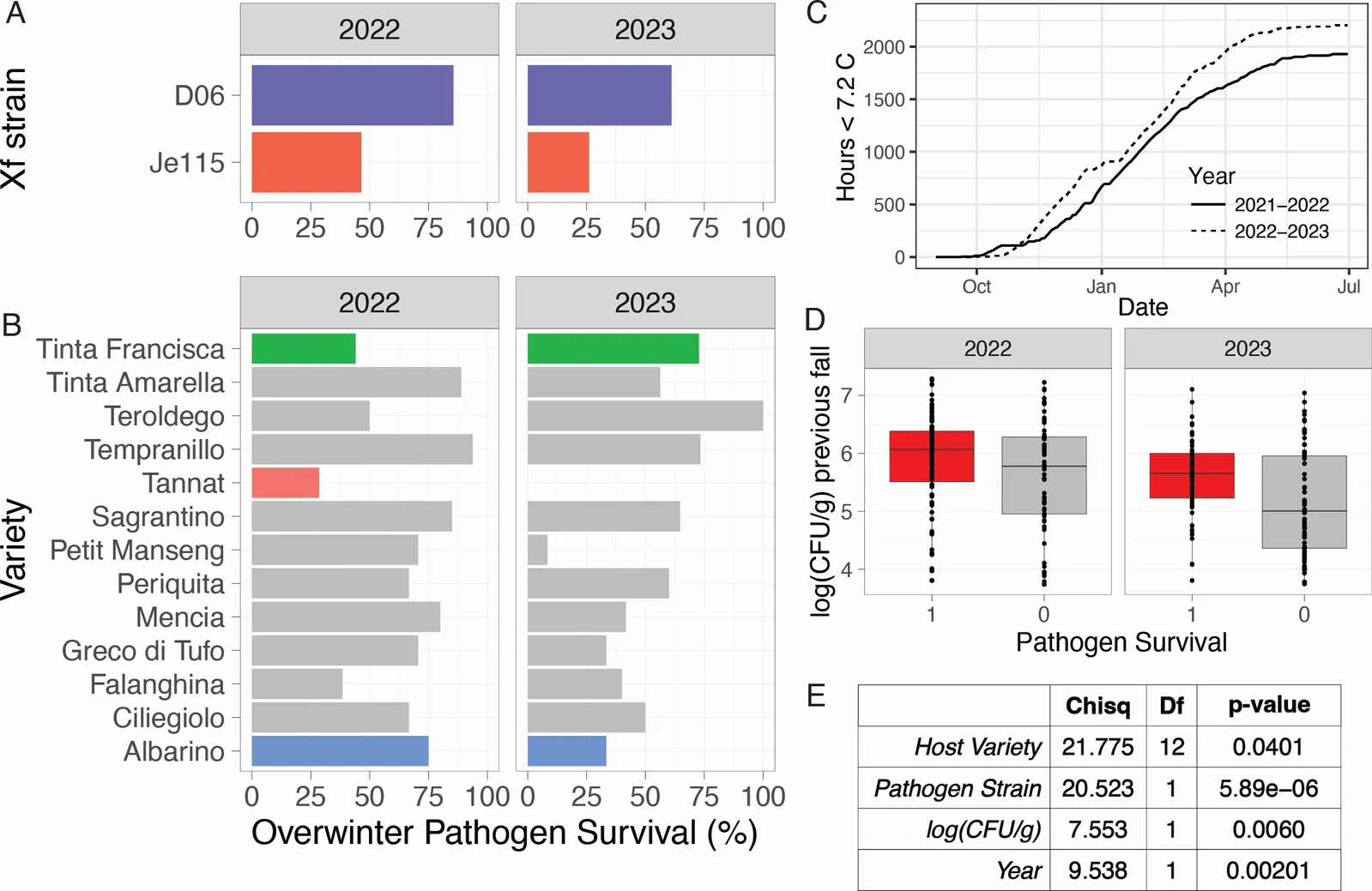
Pathogen overwinter survival by genotype and environmental factors. All *Xf* survival percentages (no. of vines *Xf* positive in current year/no. of vines *Xf* positive in previous year) are yearly, not cumulative across both years. (**A**) Pathogen overwinter survival percentages in vines infected with a local strain (D06) compared to a non-local strain (Je115) for each year of the field trial. Only vines positive for infection in the previous season were included. (**B**) Pathogen overwinter survival percentage by variety (the three varieties with non-gray colors are the same varieties with extra sampling from Fig. 3). No *Xf* infections survived the winter in Tannat vines in 2023. (**C**) Chill hour (below 7.2°C) accumulation over both winters. (**D**) Pre-winter pathogen load (log CFU/g plant tissue) in individual vines (one dot per vine) in which *Xf* infections survived (red: survival = 1) compared to infections that did (gray: survival = 0). (**E**) Analysis of deviance table from a GLMM of pathogen overwinter survival.

**Fig 5:**
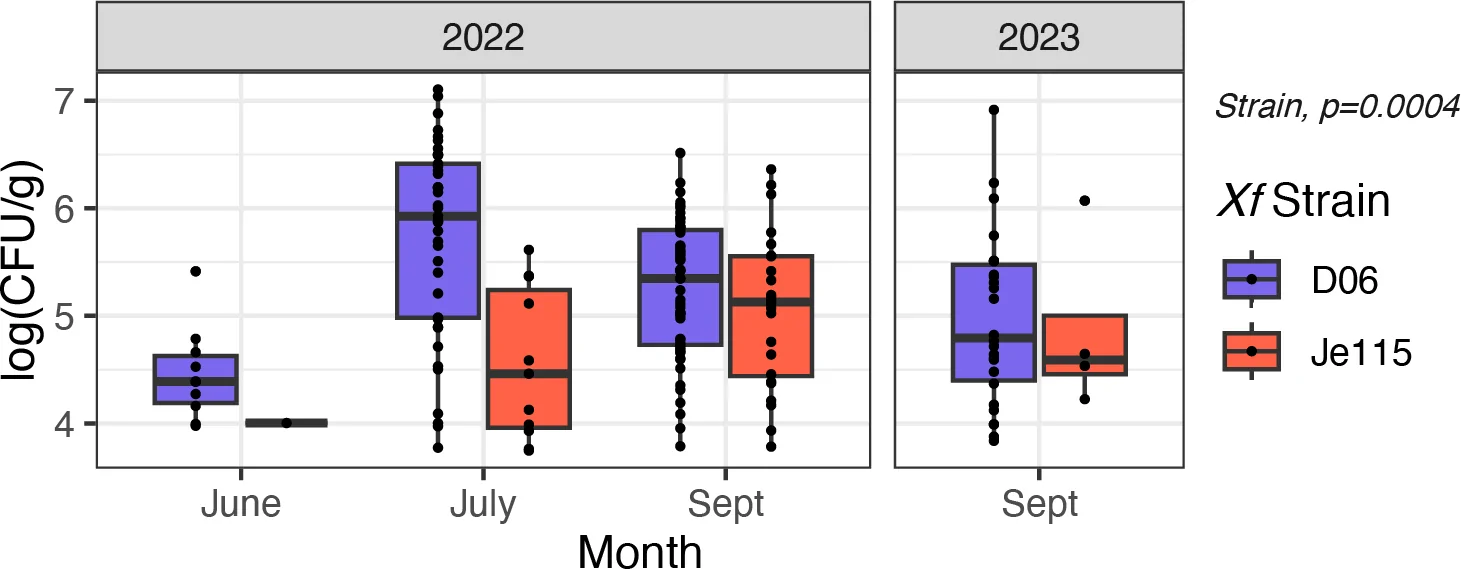
Bacterial populations vary by strain after the winter. Population estimates in 2022 and 2023 for vines that were tested in all of June, July, and September 2022 and September 2023 (*n* = 141). In 2023, qPCR results were negative for all but two vines in June and July. The *P*-value (right) is from a deviance Chi-square test from a linear mixed model.

Both biological (host and pathogen genotypes) and environmental factors (temperature) strongly influenced *Xf* overwinter survival. Across both winters, non-local Je115 strain infections survived at significantly lower (𝛸^2^ = 20.52, df = 1, *P* < 0.0001) rates (2022: 46%, 46/99, 2023: 26%, 12/46) compared to local D06 strain infections (2022: 86%, 95/111 and 2023: 61% 58/95) (Fig. 4A; Fig. S13). After the first winter, vines infected with the local D06 strain contained larger bacterial populations in 2022 than those infected with Je115 (𝛸^2^ = 12.68, df = 1, *P* = 0.0004) (Fig. 5). Overall, a cumulative pathogen survival rate of 52% for D06 and 12% for Je115 infections indicates a 77% decrease in fitness in the non-local strain.

We also measured a wide range of *Xf* survival rates across grapevine varieties (𝛸^2^ = 21.775, df = 12, *P* = 0.0401) (Fig. 4B; Fig. S13), indicating a role of grapevine physiology in pathogen winter survival. Cumulative *Xf* survival rates by variety had positive correlation coefficients (*R*) with susceptibility, but coefficients were not statistically significant (Fig. S14). Infections of larger bacterial populations (log CFU/g) in the previous year were more likely to survive the winter (β = 0.56 ± 0.20, *P* = 0.0060) (Fig. 4D). In addition to biological factors, the environment played a significant role in overwinter survival (Fig. 4C). The second winter (2022–2023) was colder than the first (2021–2022), and reflecting this, *Xf* survival rates were lower (𝛸^2^ = 9.538, df = 1, *P* = 0.0020) in the second year (50%; 70/141) compared to the first year (68%; 141/210). All four factors (strain genotype, host genotype, year, and bacterial populations) significantly impacted pathogen overwinter survival (Fig. 4E).

### Evolution of *Xf* under field conditions

**Fig 6:**
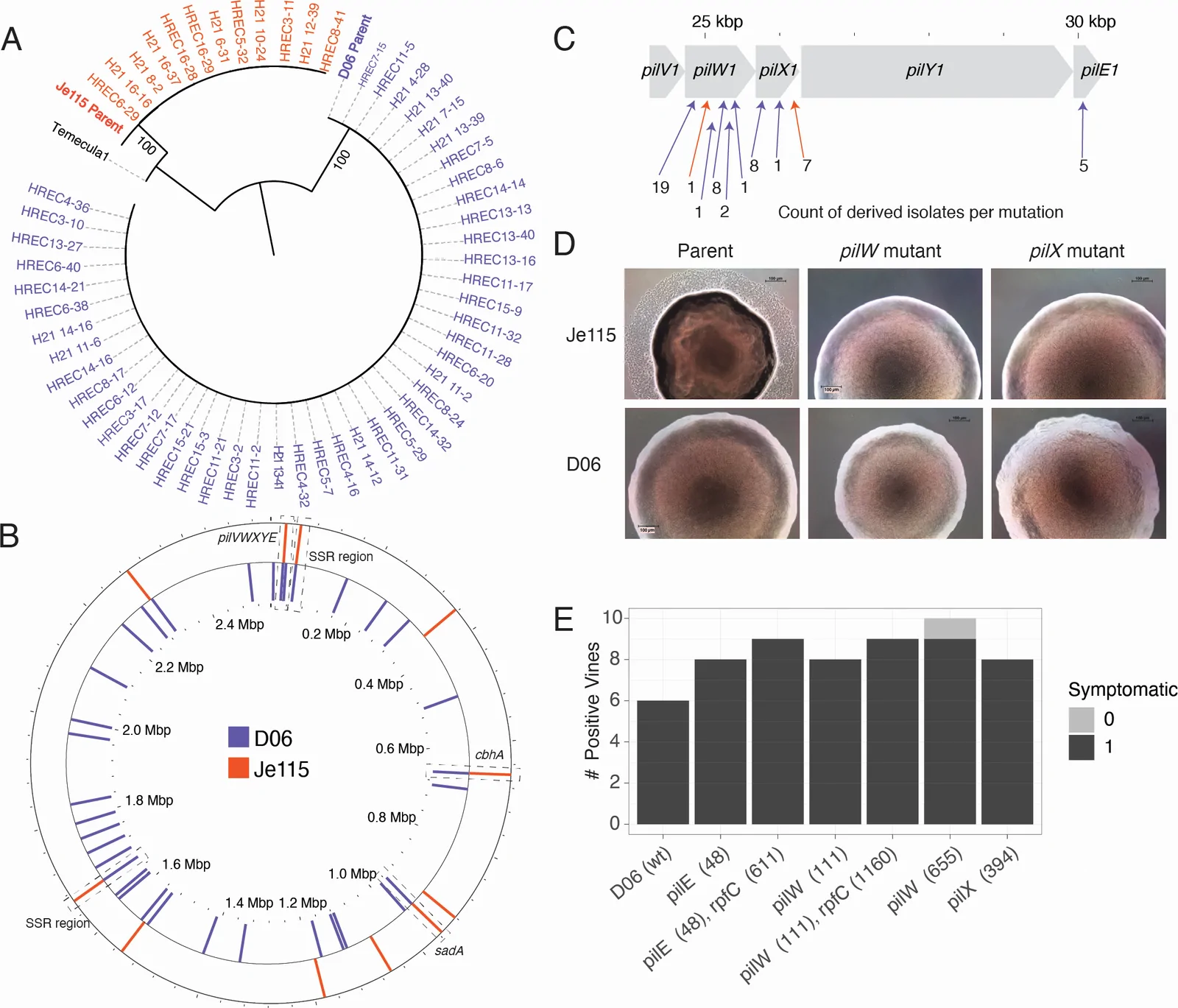
In-field evolution in two distinct *Xf* strains. (**A**) Maximum likelihood phylogenetic tree of derived isolates with two parent strains. Only solid black lines are phylogenetic branches. Strains with the prefix “H21” and “HREC” are from 2021 and 2023, respectively. (**B**) Chromosomal map of mutations found in derived isolates, mapped onto the D06 genome (2.5 Mbp). Dashed boxes show genes or SSRs with convergence between strains. (**C**) A genetic region (*pilVWXYE* operon) with convergent mutations; the arrows below the gene are color-coded based on the parent strain of the derived isolates (red: Je115, purple: D06); the counts below the arrows are the number of derived isolates containing that exact variant. (**D**) Photos of bacterial colonies in the Je115 and D06 parent strains and several derived isolates. The presence of “fringe” around colonies indicates twitching motility. Neither D06 nor any derived isolates showed any twitching motility, while the Je115 parent strain did show twitching (Table S3). (**E**) Symptom presence or absence from greenhouse experiment 2 in Cabernet Sauvignon vines. D06-derived strains are labeled with multiple distinct *pil* and *rpfC* mutations. Data from the first experiment are included in Fig. S15.

We collected “derived” bacterial isolates from individual vines infected with one of the two parent strains (D06 or Je115) after one (2021: *n* = 15) or three (2023: *n* = 43) field seasons. A maximum-likelihood phylogeny demonstrated that the derived isolates were clonal derivatives of the two parent strains (Fig. 6A). Variants (including mutations, simple sequence repeats [SSRs], and insertions/deletions) were distributed throughout the 2.5 Mbp genome (Fig. 6B). Assuming a 6-month growing season for *Xf*, the mutation rate (mutations/site/year) estimated from 2021 isolates was 1.92E10^−6^ for D06 and 1.69E10^−6^ for Je115, and from 2023 isolates 3.85E10^−7^ for D06 and 3.43E10^−7^ for Je115 (Table S6).

Convergence occurred at various levels, including genes as well as identical indels and SNPs. First, the same SSRs were repeatedly gained or lost, both among isolates from the same and from different parent strains (Fig. 6B; Table S5). All D06-derived isolates had the same synonymous mutation in autotransporter adhesin *sadA*, presumably originating from the lab stock, and one Je115-derived isolate also showed a synonymous mutation in *sadA*. Three D06-derived isolates had nonsynonymous (NS) mutations in putative transcriptional regulator *yybR*, two had NS mutations in sensory/regulatory protein *rpfC*, and three—all isolated from host variety Tempranillo—had NS mutations in glycerol-3-phosphate dehydrogenase *glpD* (Table S5). Six D06-derived isolates had the same SNP in response regulator *mprA_2*. Within Je115-derived isolates, there were three with NS mutations in response regulator *pleD_2*, with two of those mutations being identical (Table S5). One D06- and one Je115-derived isolate had NS, disruptive deletions in exoglucanase A, *cbhA* (Fig. 6B).

### Convergent mutations in Type IV pili genes suggest adaptive evolution

There was widespread convergence in mutations in a genetic operon (*pilVWXYE*) encoding minor pilins in Type IV pili (T4P) (Fig. 6C), which control twitching motility, natural competence, and other virulence phenotypes (25). Nonsynonymous variants in one of three genes (*pilW1, pilX1,* and *pilE1*) were present in 10 of 15 isolates from 2021 and 42 of 43 isolates from 2023 (Tables S3 and S5). There were 10 unique variants in these genes across 52 *pil*-mutant isolates (10 from 2021 and 42 from 2023); no isolate contained more than one distinct variant in this operon (Fig. 6C; Table S3). All 10 unique variants resulted in either a frameshift or stop-gain in the protein. Je115- and D06-derived isolates showed different genetic variants. While 6 of 10 *pilVWXYE* mutations were shared by 2–19 derived isolates, there were also four singleton variants. We checked for these mutations in the original inoculum parent stocks using both Sanger and Nanopore sequencing, but did not detect them (Fig. S5 to S7).

We then tested the two parent strains and several *pilVWXYE*-mutant-derived isolates for twitching motility *in vitro* and virulence *in planta*. Among the two parent strains, Je115 showed consistent twitching motility, while D06 did not (Fig. 6D). None of seven unique *pilVWXYE* mutants (two Je115-derived and five D06-derived) tested showed twitching motility *in vitro* (Fig. 6D and Table S3). We tested several *pilVWXYE* and *rpfC* mutants in greenhouse trials, since nonmotile strains and *rpfC* mutants are expected to be hypovirulent or avirulent (26, 27). All derived isolates tested were able to cause symptoms in grapevines (Fig. 6E; Fig. S15 and Table S3). This aligns with the non-localized virulence observed throughout the field trial; we observed symptoms in all vine quadrants, not just in the inoculated quadrant.

## DISCUSSION

Decoding complex interactions between host, pathogen, and climate factors is critical for managing and predicting future plant disease spread. Our results show that both host (grape variety) and pathogen (*Xf* strain) genotypes exert strong effects on PD susceptibility and overwinter survival of the pathogen. Using a common garden experiment *in situ*, we demonstrated a clear advantage of the local *Xf* strain in surviving cold winters, indicating local adaptation to temperature in a bacterial plant pathogen. In addition, there were convergent loss-of-function mutations in genes encoding minor T4P proteins, suggesting rapid adaptive potential. Surprisingly, a lack of twitching motility was not accompanied by a loss of virulence or localized symptoms, as observed previously in nonmotile *Xf* strains with other T4P gene mutations (25, 27).

Host and pathogen genotypes strongly influenced both PD progression and overwinter pathogen survival. Despite its importance to *Xf* spread globally, the factors determining overwinter survival are complex and poorly understood (21). *Xf* survival differences across 13 distinct varieties provide strong evidence that overwinter survival of the pathogen during cold exposure is mediated by host physiology in addition to cold temperatures, consistent with past results (11, 17). These Mediterranean varieties are cultivated primarily in Europe, where PD is an increasing concern after multiple introductions of *Xf* (28, 29). Some of these varieties are planted in CA, where PD has plagued viticulture for over a century (24). Thus, variety-specific PD susceptibilities have both applied and experimental implications. The complex interplay between temperature and host and pathogen genotypes may explain why our results differ from one past study, which did not show any effect of *Xf* genotype on pathogen survival in warmer winters (20). Potted vines in the greenhouse and cold chambers may not fully reflect the overwintering process in the field (12). Additionally, across 13 Mediterranean varieties, there was evidence that early-season stunting is an important PD symptom (Fig. S9). While stunting has been observed before in standard CA varieties (17), its occurrence in additional varieties further indicates that stunting could be a key early-season symptom of PD.

Importantly, there was evidence of local adaptation to cold winters between regional *Xf* strains in California, consistent with a previous reciprocal transplant experiment (19). As hypothesized, the local strain survived two winters at significantly greater rates than the non-local strain. Strain effects on overwinter survival were independent of bacterial population sizes, which did not differ by strain before the first winter (Fig. 1). Our data show that the local strain has adapted to survive at a higher rate over the winter, likely by resisting cold-induced host factors. Although this study includes only two *Xf* strains, the field study included a large number of vines, and differences in overwinter survivorship were consistent across 13 grapevine varieties, increasing robustness. While bacterial local adaptation to temperature is understudied (3), it is extremely relevant to forecasting the future spread of cold-limited bacterial pathogens such as *Xf*.

We also observed greater symptom incidence in vines infected with the cold-tolerant local strain. Previous work comparing these two *Xf* genotypes also demonstrated that the cold-tolerant genotype is more virulent, especially after the winter (19). This may reflect a counter-gradient variation in pathogen aggressiveness, where the genetic effects oppose environmental effects on phenotypes (30). The cold-adapted *Xf* strain may have evolved to be more aggressive during the growing season to compensate for cold-induced population decline. The tradeoff may occur if the cold-adapted strain is too aggressive in a milder winter, killing the vine (19). There are examples of counter-gradient variation in *Phytophthora* and fungal plant pathogens, where strains from colder climates show greater aggressiveness or higher thermal optima than strains from warmer climates (31, 32).

In both strains, there was convergent selection for loss-of-function mutations in minor T4P proteins, indicating adaptive evolution. Selection may not be acting on the twitching motility phenotype, but on other traits linked through pleiotropy. In the local D06 strain, where the parent strain already lacked twitching motility despite an intact *pilVWXYE* operon, these mutations still rose to high frequency in derived isolates. In addition to twitching motility, T4P also influences phenotypes like natural competence, adhesion, and biofilm formation (25). Knockouts in *Xf* of *pilE1*, *pilW1*, and *pilX1* showed a loss of twitching motility, but also decreased natural competence and increased growth *in vitro* compared to the wild-type strain; biofilm formation was increased in *pilX1* mutants (25). An increased growth rate could be advantageous in producing large bacterial populations to better survive the cold winter; in the summer, a faster growth rate would enable these mutants to outcompete others.

While these mutations were seemingly adaptive in this experiment, in nature there should be a fitness cost to nonmotility for many bacterial pathogens. Most *Xf* subsp. *fastidiosa* strains in nature retain twitching motility (33, 34), which is canonically necessary for host virulence and host colonization in *Xf* (25, 27) and in other bacterial pathogens (35, 36). Loss of twitching should impair movement against the xylem flow (basipetally i.e., upstream) but may not affect downstream (acropetal) movement (36). While we did not measure upstream movement of *pilVWXYE* mutants in greenhouse experiments, nonmotile strains moved across both vine cordons after inoculation into the Western quadrant in the field, which implies basipetal movement. This finding was surprising and requires additional research to understand when motility is and is not required during the disease cycle. The cost of *pilVWXYE* mutations may be incurred during bacterial retention in the insect vector (37) or very early plant colonization, which may differ between mechanical (as in this study) and insect inoculations.

Our results underscore the vital importance of understanding pathogen evolution in predicting global plant disease outcomes. Within a 3-year field trial, we observed widespread mutational convergence in the *pilVWXYE* operon, suggesting rapid adaptive potential of *Xf* in the field. In addition to short-term evolution, distinct *Xf* strains seem to have already adapted to local climates. The two *Xf* strains tested here are both from CA and within the same subspecies, yet showed vastly different outcomes in a colder viticulture region in CA. Many models predicting global *Xf* range or overwinter recovery from PD include environmental predictors (14, 38) but ignore pathogen genotype and adaptive potential. To accurately predict global *Xf* spread in both managed and natural ecosystems, pathogen genotype must be incorporated into models and considered carefully in experimental design.

Responses to climate change will vary across populations in a species range (39). While local thermal adaptation has been found in *Phytophthora* and fungal plant pathogens (40–43), this study demonstrates its existence in the bacterial plant pathogen *X. fastidiosa*, which has vast scientific and economic importance (44). Current local adaptation to temperature interacts with species’ potential for adaptive evolution. In models of vector-borne diseases, in addition to local thermal adaptation (45), evolutionary adaptation to high temperatures in mosquitoes can keep pace with predicted warming (46). Managing global plant disease spread will require a thorough examination of current local adaptation and future adaptive potential.

## MATERIALS AND METHODS

### Vineyard inoculation

A common-garden field experiment was performed at an experimental vineyard originally planted in 2011 at the Hopland Research and Extension Center (HREC) in Hopland, CA (Fig. 1A). The 13 European grapevine (*V. vinifera*) varieties were grafted on 101-14 rootstock in blocks of five across the plot (Fig. S1); at the start of the trial, vines were 10 years old. The selected varieties are mainly cultivated in Mediterranean areas of Europe, from Portugal to Italy. Prior to the experiment, a subset of 112 of the 477 vines (8 vines per row in 14 rows) all tested negative with qPCR for *Xf*; visual assessments corroborated no evidence of PD at the site in 2020. The vineyard was maintained by the HREC staff following standard practices for commercial vineyards in this region, including standard pesticide applications to prevent the spread of pathogens by insect vectors.

We selected two genetically distinct strains of *Xf* isolates from different California climates as strain treatments: (i) D06 (SAMN18744352), a local strain isolated from Hopland, CA, in 2020 (24), and (ii) Je115 (SAMN10358680), a non-local strain isolated from Bakersfield, CA, in 2016 (5). D06 and Je115 are phylogenetically nested within distinct PD-causing *Xf* populations from Hopland and Bakersfield, respectively (5, 24); we chose one representative strain from each phylogenetic clade. Hopland is located in Mendocino County at the Northern range edge of PD in California; the winters are colder than in the Central Valley (Fig. 1A). Bakersfield is in Kern County, at the southernmost region of the California Central Valley, with hotter summers than in Hopland. The two locations are approximately 500 km apart. Because both strains were tested on 13 Mediterranean varieties that are uncommon in California, these two *Xf* strains are not expected to have prior adaptation or specialization to any of these host varieties.

Mechanical (needle) inoculations were performed on 4 May 2021, with previously described methods (17) by inoculating two 10 µL droplets of cell suspension. Early May is when natural, vector-borne infections are expected to occur and result in chronic infections (13). Strains were grown from freezer stocks and maintained in the lab on PD3 plates (47) in late March and April 2021. For each variety, there were 10 negative controls (mock-inoculated), and the remaining vines (*n* = 23–30) were split between the two *Xf* strains. Treatments were randomized throughout the vineyard (Table S1). In total, there were 477 vines inoculated: 174 with *Xf* Je115, 174 with *Xf* D06, and 129 negative control vines (Table S1). Vines were trained with bilateral cordons and inoculated at the basal third of the distal-most shoot on the Western cordon (Fig. 2B). Because of the large number of vines, we prepared multiple cell suspensions for each strain to limit the loss of cell viability during hours of plant inoculations. For each suspension, a 10 µL aliquot was saved for DNA extraction and quantification; all inoculation cell suspensions (*n* = 8 for Je115 and *n* = 6 for D06) contained over 10^9^ CFU/mL. A team of five individuals performed the inoculations in 1 day in a randomized manner; there were no differences in infection rates among individuals.

### Data collection

We collected (i) petioles (the stalk between the stem and leaf) for pathogen detection, (ii) petioles for cross-vine pathogen movement in 3 of 13 varieties, (iii) symptom assessments, (iv) petioles for bacterial culturing, and (v) local temperature data in Hopland. Figure S2 depicts our data collection pipeline.

#### Pathogen detection

For all vines, including controls, in September 2021 (4 months post-inoculation), 2022, and 2023, we sampled petioles on the inoculated western (W) shoot; all were qPCR tested (see “qPCR methods for field and greenhouse experiments,” below). In 2022 and 2023, we collected W petioles in both June and July from vines that had tested positive in the previous year. On the final sampling date (September 2023), we also took additional petioles from symptomatic tissue (any quadrant) for vines that tested positive in 2022.

#### Cross-vine pathogen movement

To assess pathogen movement over all years (2021–2023), we collected petioles on the West (W; where infection occurred), West-Center (WC), East-Center (EC), and East (E) quadrants for three varieties (Albariño, Tannat, and Tinta Francisca) in June, July, and September. Only 3 of 14 varieties were selected for cross-vine sampling to reduce the number of DNA extractions and qPCRs. We only tested WC, EC, and E samples for vines that were positive in the W quadrant in that specific month and year.

#### Symptom evaluation

We evaluated symptoms for all vines in August and September of each year. Symptoms were scored by quadrants (W, WC, EC, and E) as binary (1/0) for the following categories: leaf chlorosis, leaf scorch, matchstick petioles, stunting, shriveled berries, uneven cane maturation, barren cane (2021 only), and dead cordon (2023 only). In both April and May 2022 and 2023, we assessed early-season stunting by measuring three shoots per vine on the W quadrant.

#### Bacterial culturing

We re-isolated the two *Xf* strains from the field in September 2021 (48 vines) and in September 2023 (116 vines). Standard methods were used to culture *Xf* on PD3 solid media plates (47), and each strain was triple-cloned before DNA extraction. All viable strains were frozen in 30% glycerol stocks. We acquired 15 derived isolates (nine D06 and six Je115) in 2021 and 43 isolates (37 D06, 6 Je115) in 2023.

#### Temperature data

Chill hour (<7.2°C/45°F) accumulation is a standard metric in assessing winter severity in agriculture generally and for PD overwinter recovery specifically (12). We downloaded chill hour data from 1991 through 2026 from the Sanel Valley station (<1.5 miles from the HREC experimental vineyard) and from the Arvin-Edison Station in Bakersfield from the UC Davis Fruit & Nut Research & Information Center (48). Additionally, we logged temperature hourly with Hobo Data Loggers during the winter months (October through April) from 2022 to 2025 in both locations: Hopland Research and Extension Center in Hopland and the University of California Cooperative Extension Office, Kern County in Bakersfield. From this data set, we calculated freezing (<0°C) and chill hours cumulatively in each location (Fig. S16).

### Data cleanup

Each year, we tested all 129 control plants in September. Only one control vine tested positive (in both 2022 and 2023); based on strain-specific primers (Donegan, unpublished data), we determined it was strain D06 and presumed it was a vine inoculated by mistake. The annual negative results in 128 of 129 control vines indicate no spread of *Xf* within or into the vineyard. Using a histogram of the control vines, we determined a CT cutoff of 37 for qPCR (Fig. S3). Vines with missing samples in any year (*n* = 15) were removed from the data set, except for a vine that was positive in 2021 and 2022 but dead in 2023. In 2023, there was no growth on the vine, so we were unable to sample for qPCR. We presume this vine was still infected in 2023.

We determined infection status by year (2021, 2022, and 2023) as 1/0 for all vines (Table S1). If a vine had a positive sample (by culturing or qPCR) at any time point in that year, it was coded as *Xf* positive. We presumed that vines positive in a later year (2022 or 2023) that tested negative the previous year were positive in the previous years (2021 and/or 2022). That is, we determined that these samples were most likely false negatives in qPCR testing, rather than new infections. There was an estimated false negative rate of 19.4% (41/211) in 2021 (the year of infection) and 5% (7/142) in 2022 (Fig. S4); false negatives are likely due to heterogeneity of *Xf* populations within plant tissues.

### Data analyses

We built several linear or generalized linear models (LM/GLM) and mixed models (LMM/GLMM) to analyze our data sets. Unless otherwise noted, all negative control vines were excluded from statistical analyses. For GLMMs and LMMs, we performed a Wald’s Chi-square test or deviance Chi-square, respectively, to test for significance of fixed effects.

#### Infection success in year 1

Using the cleaned data with inferred positives, we built a binomial GLM with infection status (1/0) in 2021 as the response and variety and strain as predictors.

#### Bacterial population sizes before the first winter

Estimates of populations were included from vines that tested positive by qPCR in September 2021. The data set was approximately normally distributed. We then built a linear model with bacterial populations (calculated from qPCR CT values) as the response, and variety and strain as predictors.

#### Second season bacterial population sizes

For this data set, we only included vines (*n* = 141) that were tested in all months (June, July, and September) of 2022, since in 2023, there were only positives in September. We used a linear mixed model to analyze bacterial populations in 2022 as a function of variety, strain, month, and strain × month, with a random effect of vine identity.

#### Cross-vine movement

The qPCR data set (not including inferred positives) was subset to only include Albariño, Tinta Francisca, and Tannat samples from all quadrants from September (all 3 years). For 2021, the EC and E quadrant samples were not tested because all WC samples were negative; inferred negatives (0 infection status) were added for the EC and E quadrants for September 2021 to all vines. The GLMM had infection status (1/0) as a response, with strain, variety, quadrant, year, and a quadrant × year interaction term as fixed effects and vine identity as a random effect.

#### Pathogen overwinter survival

*Xf* survival was coded as a binary (1/0) for each vine that was positive in the previous year at any time point. We only included vines that had a positive qPCR result at some point in the trial, so that we could include the maximum pathogen populations in plants (from qPCR CT values) from the previous year in the model. Vines that were never positive were excluded. There were two data points for each vine (overwinter survival in 2022 and 2023), except for vines for which that pathogen did not survive from 2021 to 2022. We built a binomial GLMM with pathogen survival (1/0) as the response and year, variety, strain, and the maximum pathogen populations in the previous year as fixed effects; vine identity was included as a random effect. All predictors were categorical variables except pathogen populations.

#### Symptom incidence before the first winter

For 2021, we included only positive vines and grouped symptoms across both August/September (one for symptoms at any time point). There were only symptoms in the Western quadrant in 2021, so other quadrants were excluded. Symptoms (1/0) were modeled in a binomial GLM with variety and strain as predictors.

#### Symptoms across the vine

We included symptoms from August and September from all quadrants from only positive vines, excluding vines that were no longer infected. We modeled symptoms (1/0) as a binomial GLMM, with variety, strain, month, year, quadrant, and year × quadrant fixed effects and a random effect of vine identity.

#### Stunting after the winter

Vine shoot stunting is a poorly characterized, early-season PD symptom (17). For each time point (April or May in 2022 or 2023), heights were averaged across the three measured shoots for each vine by quadrant. Then, we joined these data with positives for all years (included tested and inferred positives). For each year and month, we calculated the average height in uninfected plants (both controls and negative inoculated vines) for each of the 13 varieties. These values were used to normalize the measured heights for each vine. We modeled normalized height in an LMM as a function of vine status (positive/negative), strain, year, month, and variety; vine identity was included as a random effect.

#### Correlation testing

We aggregated the three susceptibility metrics in vines across the 13 varieties in 2021: (i) infection success, (ii) bacterial population sizes, and (iii) symptom incidence among positive vines. These aggregated values were correlated using three separate Pearson’s correlation coefficient tests. We also correlated these three susceptibility metrics with overall (cumulative over 2 years) pathogen survival rates in the 13 varieties.

All formulas and results from statistical models are included in Table S2.

### Derived isolate sequencing and SNP calling

We whole-genome sequenced 58 derived isolates from the field site (Table S3). Paired-end Illumina sequencing data were generated using a NovaSeq X at QB3 Genomics, UC Berkeley, Berkeley, CA, RRID:SCR_022170; de-multiplexed raw reads were returned. All raw sequencing data are available on NCBI (Bioproject: PRJNA1391514). All reads were deduplicated with bbmap (49), and adapters were trimmed with cutadapt (50). Trimmed reads were then mapped to a reference genome to call SNPs with snippy (51) with default parameters and minimum coverage set to 20. All strains (*n* = 58) were mapped to the *Xf* reference genome Temecula1 (SAMN02603844); the resulting SNP data set of all strains mapped to Temecula1 was used to build a maximum-likelihood phylogenetic tree with RAxML (52), visualized with Interactive Tree of Life (53).

We built hybrid genome assemblies of the two parent strains (D06 and Je115). Whole-genome sequencing was performed by Plasmidsaurus using Oxford Nanopore Technology with custom analysis and annotation. Using previously obtained Illumina short-read sequencing, we constructed hybrid assemblies with Unicycler v. 0.5.0 (54) by inputting the deduplicated and trimmed paired-end Illumina reads as well as the raw Nanopore reads. Known contaminant sequences (i.e., PhiX bacteriophage) and contigs less than 200 bp were removed. For both hybrid assemblies, we constructed annotations using Prokka v. 1.12 (55) and checked genome quality using Quast 5.0.2 (56). The D06 genome assembly contains a single, circular chromosome (2.5 Mbp) with no plasmids. The Je115 genome assembly includes a chromosomal contig (2.5 Mbp), a 38.3 kbp plasmid, and 12 small (<2,000 bp) fragments. All genome quality statistics are listed in Table S4. Nanopore data for hybrid assemblies are publicly available at NCBI (D06, SAMN18744352; Je115, SAMN10358680).

Then, all derived isolates were split into groups by parent strain (D06/Je115) and mapped to the hybrid genome assembly of the parent strain. Based on the number of SNPs when mapped to the parent strain and a phylogenetic tree created with RAxML, all parent strains were concordant with the known inoculated strain for each vine (Fig. 5A). We combined the snippy outputs of each derived strain mapped to the respective parent strain to examine insertions, deletions, and mutations (Table S5). These variants were visualized in Proksee (57).

We calculated an in-field mutation rate (mutations/site/year) for 2021 and 2023 strains (Table S6) based on a 2.5 Mbp genome. We removed two SNPs in D06-derived strains (positions 928213 and 1413497) because they were present in all D06-derived strains, and therefore these SNPs likely occurred before the field trial. We left in non-unique SSRs, since there is evidence that SSRs are repeatedly gained and lost in bacterial field strains (58). We compared calculations defining a “growing year” in two ways: (i) actual years in the field (0.4 for 2021 strains and 2.33 for 2023 strains) and (ii) growing seasons in the field, based on a 6-month growing season from 1 April to 1 October for Mendocino County (0.75 for 2021 strains and 2.92 for 2023 strains). While these two methods have different assumptions about when *Xf* is active throughout the year, the estimates were very similar (Table S6), and we opted for the growing season calculation.

### Derived isolate mutation validation

After noticing identical mutations in the *pilVWXYE* operon in multiple derived isolates (Fig. 5), we validated these variants. First, we amplified the *pilW* and *pilX* genes in DNA extractions from six derived isolates (Table S3), freezer stocks of the two parents, and five of six D06 field inoculum stocks (i.e., DNA from the cell suspensions used for infecting the vineyard in May 2021; the DNA extraction from the last stock had been used) (primers; Table S7). Sanger sequencing was performed at the UC Berkeley DNA Sequencing Center; data were analyzed in Geneious Prime v. 2023.2.1 (http://www.geneious.com/). All sequencing results confirmed the expected mutations in derived isolates, while neither the two parent strain stocks nor the D06 inoculum stocks showed any mutations in these two genes (Fig. S5).

We then amplified part of this operon from *fimT1* to *pilX1* (2.7 kbp) in five of six of the original D06 inoculation extractions and seven of eight Je115 inoculation extractions (the missing stocks had been used at an earlier date) (primers *pil_op-F* and *pilX1-R*; Table S7). Premium PCR sequencing was performed by Plasmidsaurus using Oxford Nanopore Technology with custom analysis and annotation. None of the consensus sequences showed any mutations; they were all 100% identical (Fig. S6). We compared error rates at five sites with recurring mutations to baseline rates of error in mutation, deletion, and insertion across the entire 2.7 kbp fragment. The very low (<2%) mutation, deletion, and insertion rates observed at sites of recurring mutations were well within the error range observed across the entire amplicon (Fig. S7). In conclusion, the recurrent mutations were not detectable in the inoculum stocks from the start of the field trial.

### Twitching motility assay

A subset of the derived strains with distinct *pilVWXYE* operon mutations was tested for twitching motility. We tested the two parent strains (D06 and Je115) as well as 2 Je115-derived isolates and 5 D06-derived isolates with distinct *pilWXYE* mutations (Table S3). Twitching was assessed using previously described methods (59). Briefly, strains were retrieved from −80°C glycerol stocks and grown on PW plates for 7 days, then restreaked onto fresh PW plates and grown for an additional 7 days. Bacteria from the second streaked plate were used to make 10–12 spots on PW plates lacking BSA, and the bacterial spots were grown for 5 days; photos were taken daily to measure fringe width in microns. The presence of a fringe around colonies indicates twitching motility. The experiment was performed twice.

### Greenhouse testing of derived isolates

To validate the virulence of *pilWXYE* mutants, we performed two independent experiments of greenhouse inoculations at the Oxford Tract Greenhouse at UC Berkeley. Experiment 1 was performed on cv. Ciliegiolo vines; plants were inoculated on 10 January 2024 and kept in a greenhouse with supplemental lighting until the end of January 2025. We mechanically inoculated (17) five plants per treatment, including a control (mock inoculation), parent/wild-type D06, parent/wild-type Je115, and six derived isolates (Table S3) for a total of 50 plants. Symptoms (1/0) were evaluated at 8 and 14 weeks after inoculation; petiole samples for qPCR were tested at 14 weeks (Fig. S15). Experiment 2 included 75 cv. Cabernet Sauvignon vines; five control plants and 10 plants per strain, including parent/wild-type D06 and 6 D06-derived strains (Table S3). All plants were mechanically inoculated on 5/30/25. Symptoms were evaluated at 9 and 12 weeks, and qPCR was performed at 12 weeks.

### qPCR methods for field and greenhouse experiments

DNA from plant tissue and bacterial cells was extracted using a DNEasy Plant Mini Kit (Qiagen) and DNA Blood and Tissue Kit (Qiagen), respectively. For all plant samples, we used 0.1 g combined from two petiole samples for tissue homogenization. All qPCR testing from samples collected between 2020 and 2024 was done on an Applied Biosystems 7500 Fast Real-Time PCR System machine with previously described *recF1* primers and thermocycling conditions (60) (Table S7). The CT cutoff for all plates run on this machine was 37 (Fig. S3). For the 2025 greenhouse experiment, plates were run on a Thermo Fisher Scientific QuantStudio 3 Real-Time PCR instrument. Thermocycling conditions used were a 2-min denaturing step at 98°C, 40× cycles of 98°C for 0:15 s and 60°C for 1:00 min, followed by a dissociation curve. Due to increased sensitivity, the CT cutoff for all plates run on this machine was reduced to 32 (Fig. S8). CT values were converted to bacterial population estimates, i.e. log(CFU/g) based on empirically determined standard curves for each machine (Fig. S8).

### Software and computational resources

Bioinformatic analyses for genomic data sets were performed using the Savio computational cluster resource provided by the Berkeley Research Computing program. Code for the processing and analysis of field trial data is available on GitHub at https://github.com/MonicaDonegan/Overwinter_Adaptation. Data cleanup and analysis were performed in R, using the following packages: *stringr* (61), *dplyr* (62), *data.table* (63), and *tidyverse* (64). We also used R packages *patchwork* (65), *ggplot2* (66), *ggpattern* (67)*,* and *ggpubr* (68) for data visualization. In addition to R base statistics, we employed *lme4* (69), *glmmTMB* (70, 71), and *car* (72) packages. Figures were composed and edited in Adobe Illustrator.

## Data Availability

All raw sequencing data from derived isolates are available on NCBI (Bioproject PRJNA1391514). Nanopore data for hybrid assemblies for D06 and Je115 are publicly available at NCBI (D06, SAMN18744352; Je115, SAMN10358680). Code and data sets for the processing and analysis of field trial data, as well as hybrid assemblies of D06 and Je115, are available on GitHub at https://github.com/MonicaDonegan/Overwinter_Adaptation.

## SUPPLEMENTAL MATERIAL

Supplemental figures aem.01520-26-s0001.pdf Fig. S1 to S16.

Supplemental tables aem.01520-26-s0002.xlsx Tables S1 to S7.

ASM does not own the copyrights to Supplemental Material that may be linked to, or accessed through, an article. The authors have granted ASM a non-exclusive, world-wide license to publish the Supplemental Material files. Please contact the corresponding author directly for reuse.
