# Supplementary material for: Pathogen local adaptation shapes Pierce’s disease of grapevines outcomes under field conditions
Source: Appl Environ Microbiol. 2026 Sep 16;92(10):e01520-26. doi: 10.1128/aem.01520-26 (PMC13688793; doi:10.1128/aem.01520-26)

Figure list

S1 - Map of blocked host varieties in field plot
S2 - Diagram of field plot sampling
S3 - CT cutoffs in qPCR results from field trial
S4 - False negatives
S5 - Sanger Sequencing of *pilW* and *pilX* genes
S6 - Nanopore sequencing of inoculum stocks - consensus
S7 - Nanopore sequencing of inoculum stocks - error rates
S8 - qPCR methods and standard curves
S9 - Correlation between Susceptibility metrics in 13 varieties
S10 - Distinct Pierce's disease symptoms
S11 - Symptoms on the Western Quadrant
S12 - Stunting Measurements
S13 - Positive Vine Counts in Field Experiment
S14 - Correlation between Susceptibility and Overwinter Survival
S15 - Greenhouse Experiment #1 with Derived Isolates
S16 - Temperature data from Hopland and Bakersfield

Figure S1 Map of blocked host varieties in field plot.

Vineyard at the Hopland Research and Extension Center (HREC). Vines that did not have sufficient shoot growth for inoculations in May 2021 were excluded from the trial. Touriga Nacional was excluded because only 9 vines remained in 2020, whereas the other 13 varieties retained between 33-40 vines. All vines are grafted on 101-14 rootstock, so the only differences among varieties are related to scion material selection.

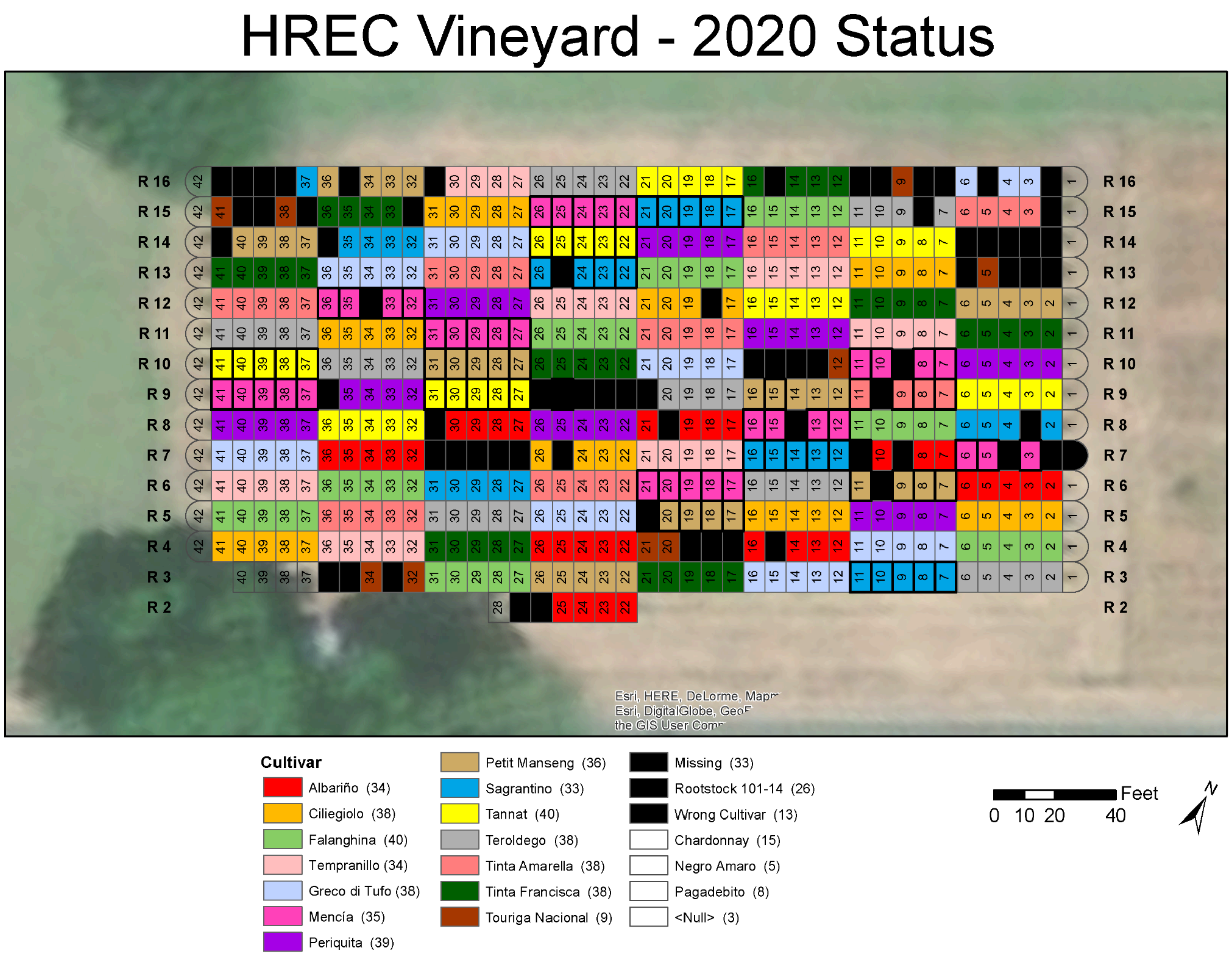

Figure S2 Diagram of data collection in three year field trial

In September of each year, we sampled petioles from all 477 vines. In 2021 in June and July, we only collected from all quadrants for three varieties (Tannat, Tinta Francisca, Albariño). In 2022 and 2023 in June and July, we collected from all quadrants for those three varieties as well as the W quadrant for vines that were positive the year before. Overall, across this field project we collected >4,000 petioles and tested 2,717 with qPCR: 698 in 2021, 1,006 in 2022, and 1,013 in 2023. Samples from the WC, EC, or E quadrants were not tested if the W sample was negative in that year and month. In July of 2023, only 2 of 166 vines were positive, so we did not test all of the June samples (only 65 were tested; all were negative).

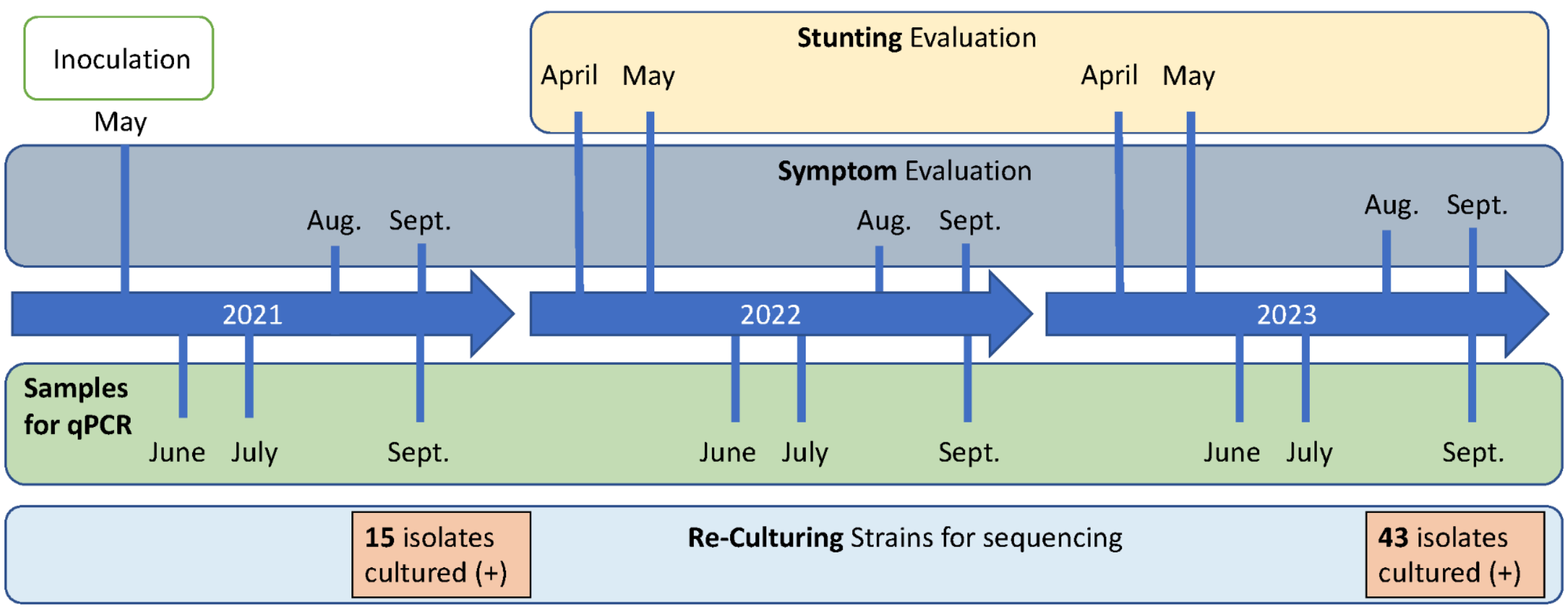

Figure S3 CT cutoffs in qPCR results from field trial

The CT distribution of negative control plants and plants determined to be negative was similar, using a CT cutoff of 37. All negative control samples had CT values above 37. The positive samples ranged from CT values of 22 to < 37 due to the cutoff.

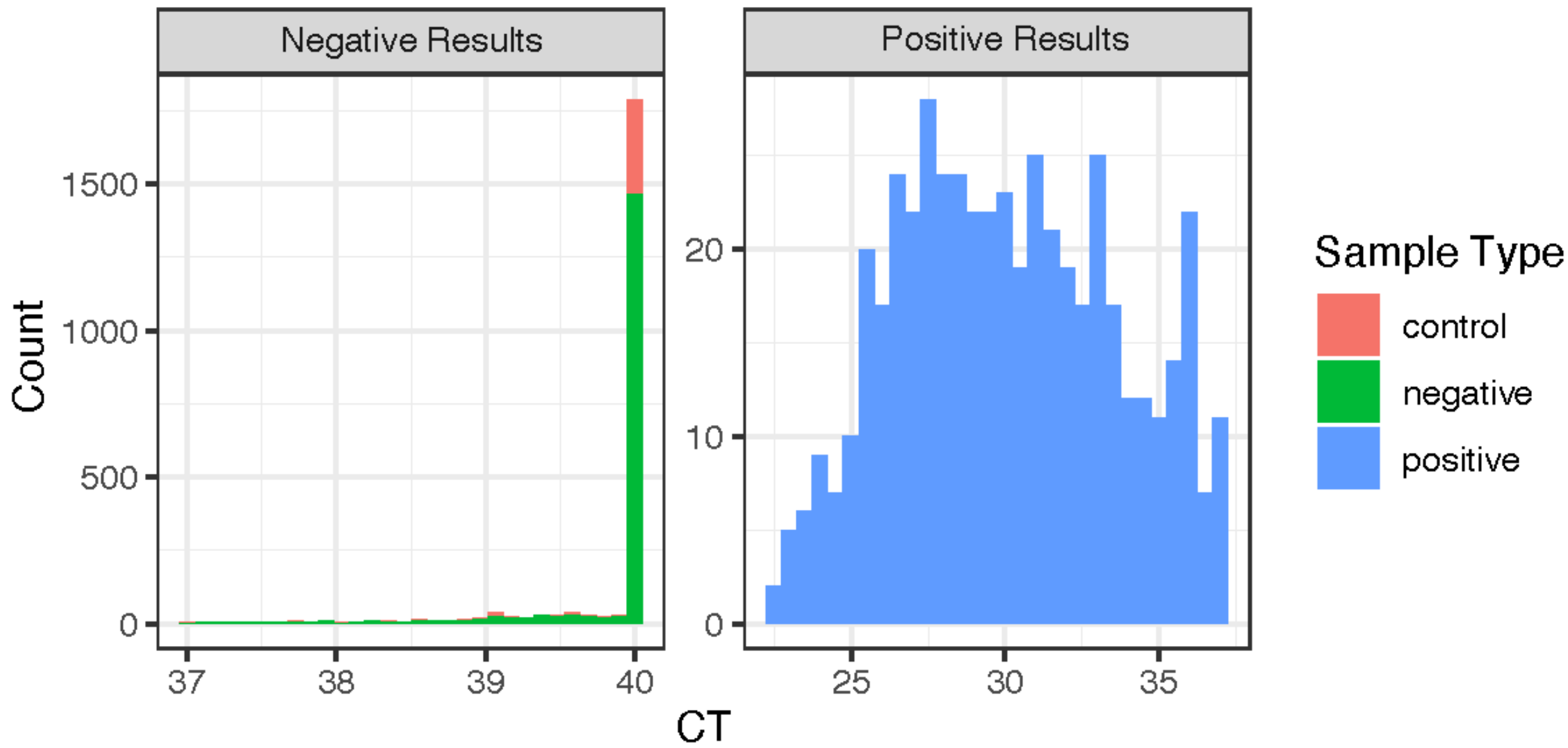

Figure S4 False negatives

Based on positive test results from later years (2022 and/or 2023), there were inferred positives / assumed false negatives in this study. (A) The distribution of false negatives was spread out across the 13 varieties in the trial. (B) The counts of inferred positives were distributed across both strains (Je115 and D06).

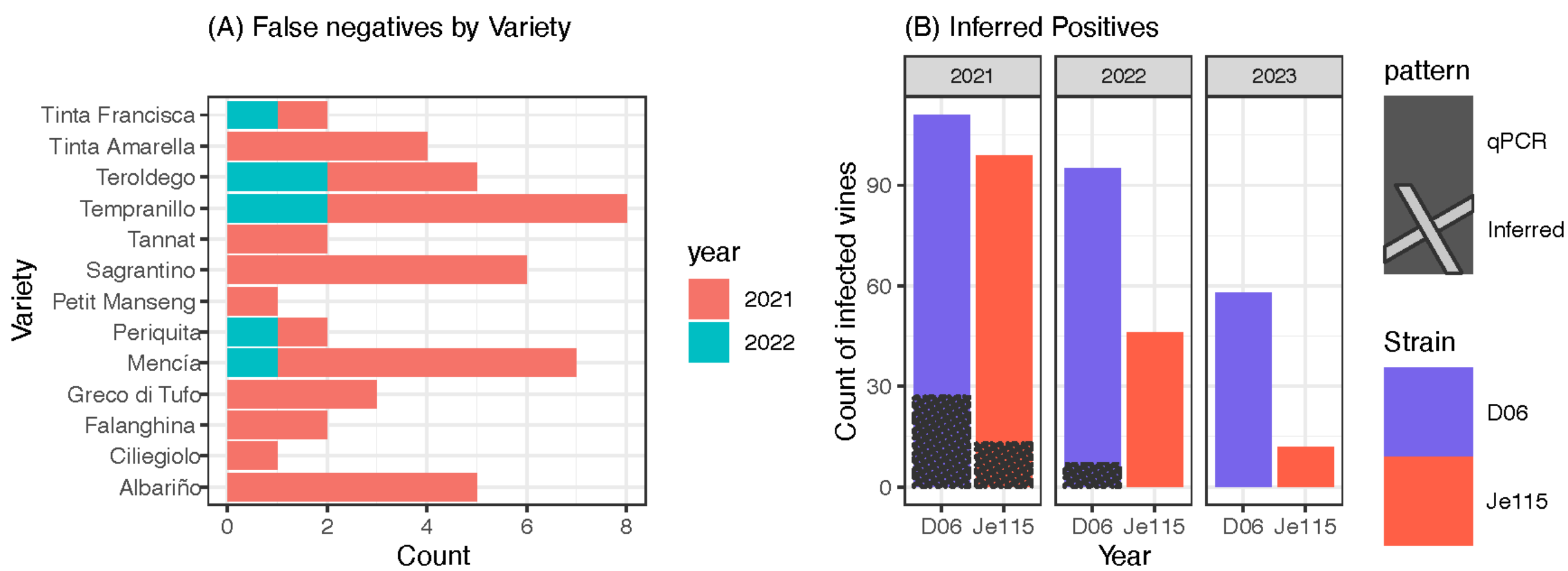

Figure S5 Sanger Sequencing of *pilW* and *pilX* genes

We aligned the sequences of *pilW* and *pilX* from (#1) the D06 hybrid assembly with Sanger sequencing consensus sequences from (#2) D06 freezer stock (#3-7) D06 inoculum extracts from the field trial (#8) Je115 freezer stock and (#9-14) Je115 and D06 derived isolates with distinct *pilW* and *pilX* mutations. For each of the mutants tested, the expected mutations are detailed in the name. The top panel shows three regions in *pilW*: (1) positions 110-120, where 19 (2 shown) D06-derived isolates had a deletion (2) pos. 230-245, where one (shown) Je115-derived isolate had a large deletion (3) pos. 650-660, where 8 (2 shown) D06-derived isolates showed a C → T substitution. The bottom panel shows 2 regions in *pilX*: (1) pos. 390-400, where 8 (0 shown) strains D06-derived isolates had a C → T substitution (2) pos. 520-530, where 7 (1 shown) Je115-derived isolates had an A insertion. Importantly, these mutations were not observed in the freezer stocks, inoculum stocks, or other mutants except when predicted.

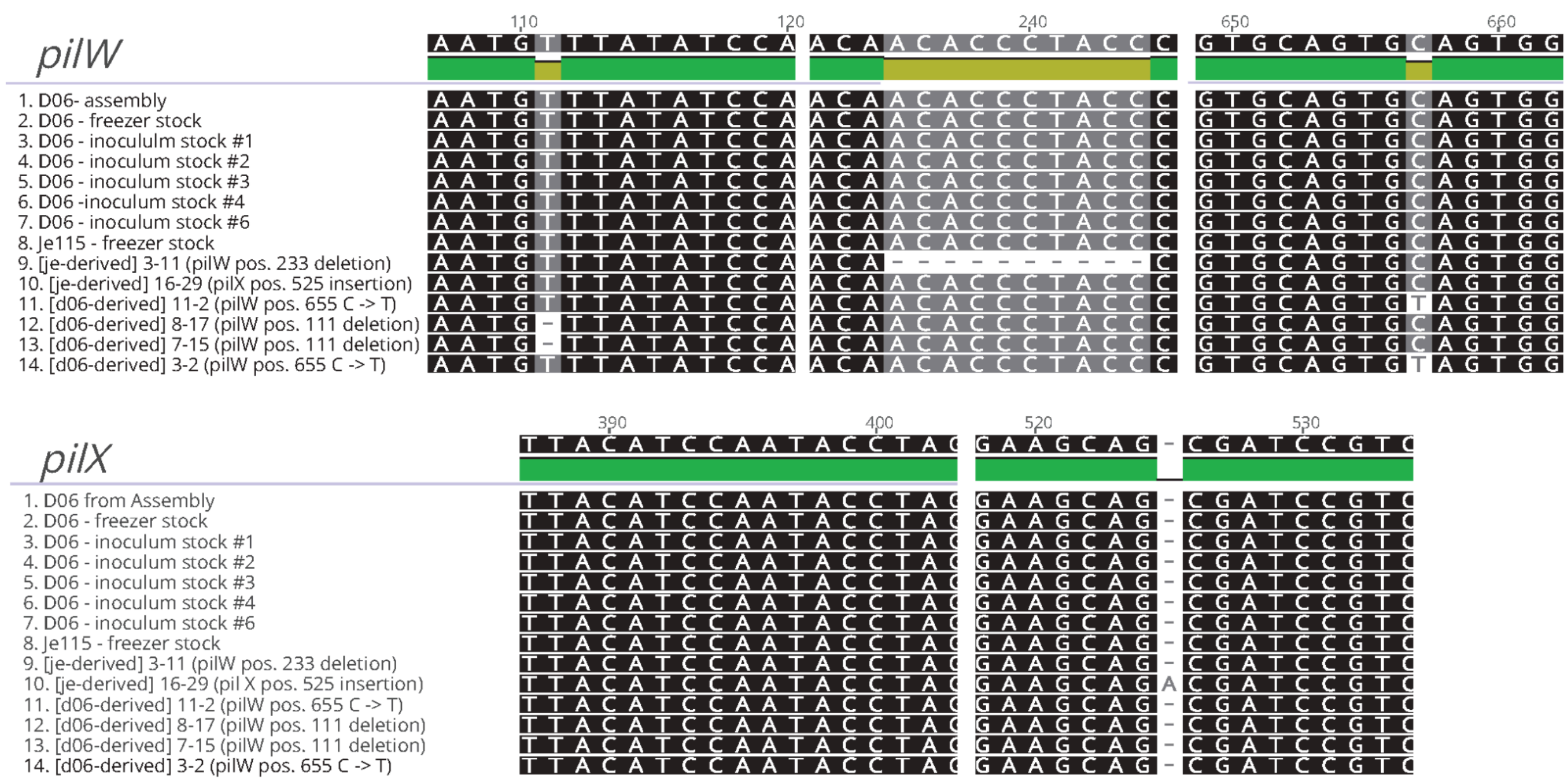

Figure S6 Nanopore sequencing of inoculum stocks - consensus

There were no mutations in *pilW* or *pilX* in any of the 6 D06 or 7 Je115 inoculum stocks, when this amplicon was Nanopore sequenced.

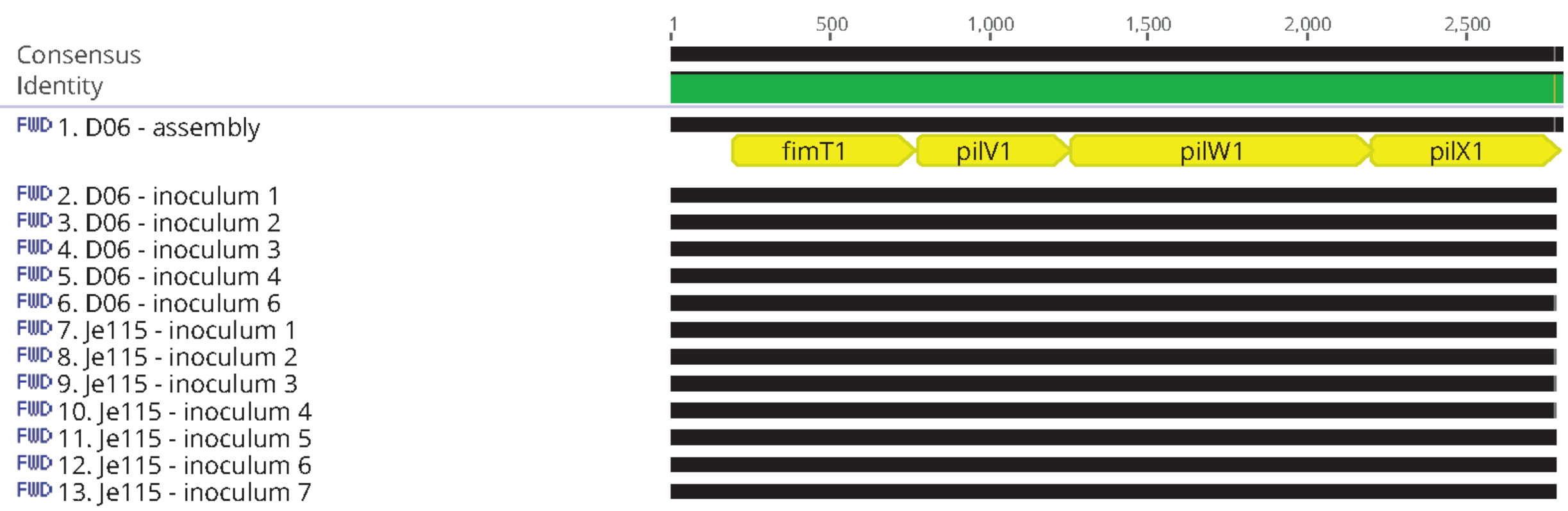

Figure S7 Nanopore sequencing of inoculum stocks - error rates

For the 6 D06 and 7 Je115 inoculum stocks tested, Nanopore sequencing coverage ranged from 60X to 1000X. Plots of mismatch, deletion, and insertion sites at every position across a 2.7 kbp amplicon from inoculation stocks. Blue dots are "background" error rates for all positions in the fragment. Red dots show the positions where shared (>1 derived isolate) mutations, deletions, or insertions were detected in the derived isolates (but not these inoculum stocks). As shown, the rates of error at these sites in the inoculum stocks (red dots) were all <2% and within background variation (blue dots). In the derived isolates, there were 3 shared mutations (mismatch) and 1 deletion in D06 derived isolates, and 1 shared insertion in Je115 derived isolates.

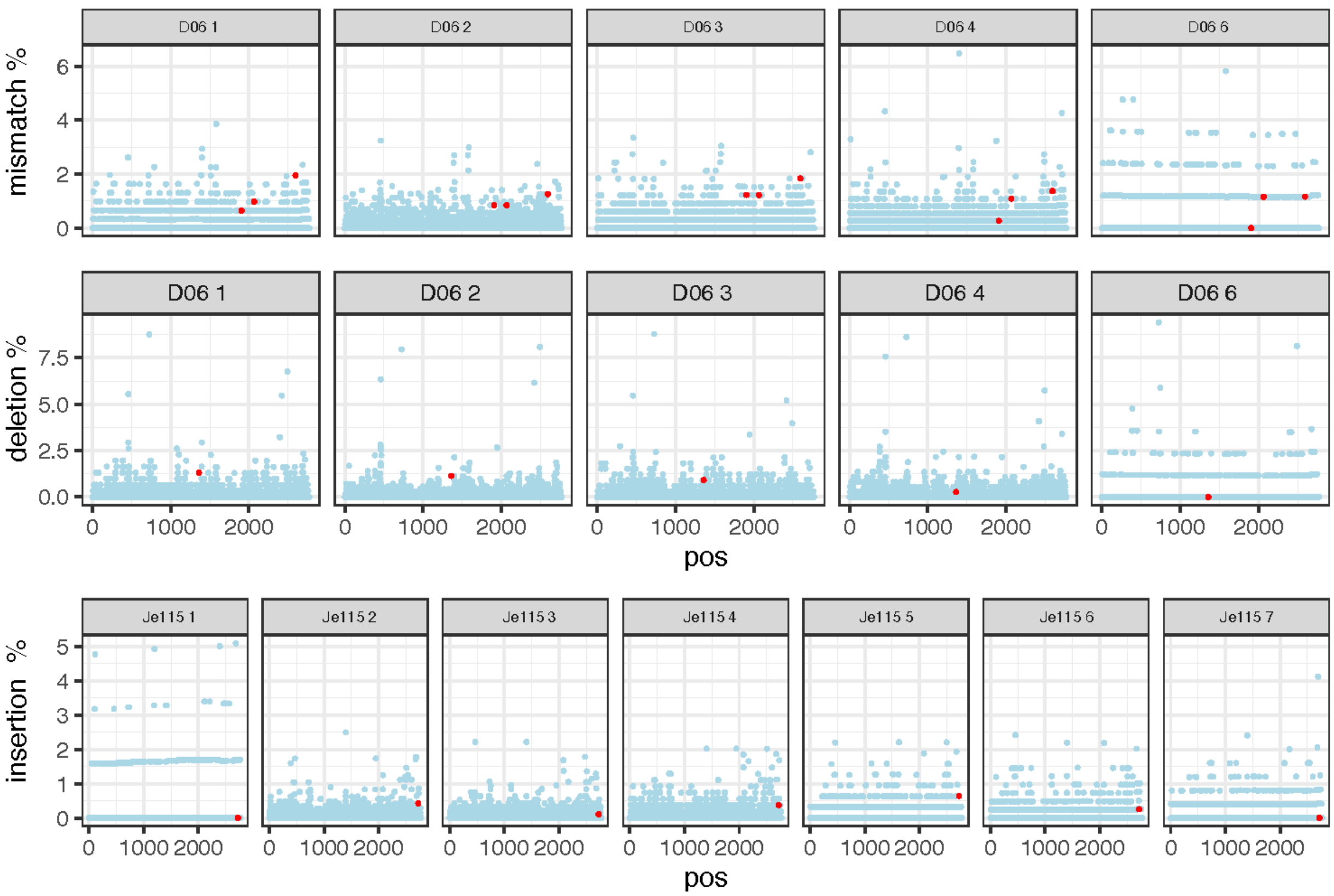

Figure S8 qPCR methods and standard curves

All qPCR testing from samples collected between 2020 and 2024 was done on an Applied Biosystems 7500 Fast Real-Time PCR System, and for the 2025 greenhouse experiment, plates were run on a Thermo Fisher Scientific QuantStudio 3 Real-Time PCR instrument. CT values were converted to bacterial population estimates *i.e.* log10 (CFU/g) based on empirically determined standard curves for each machine.

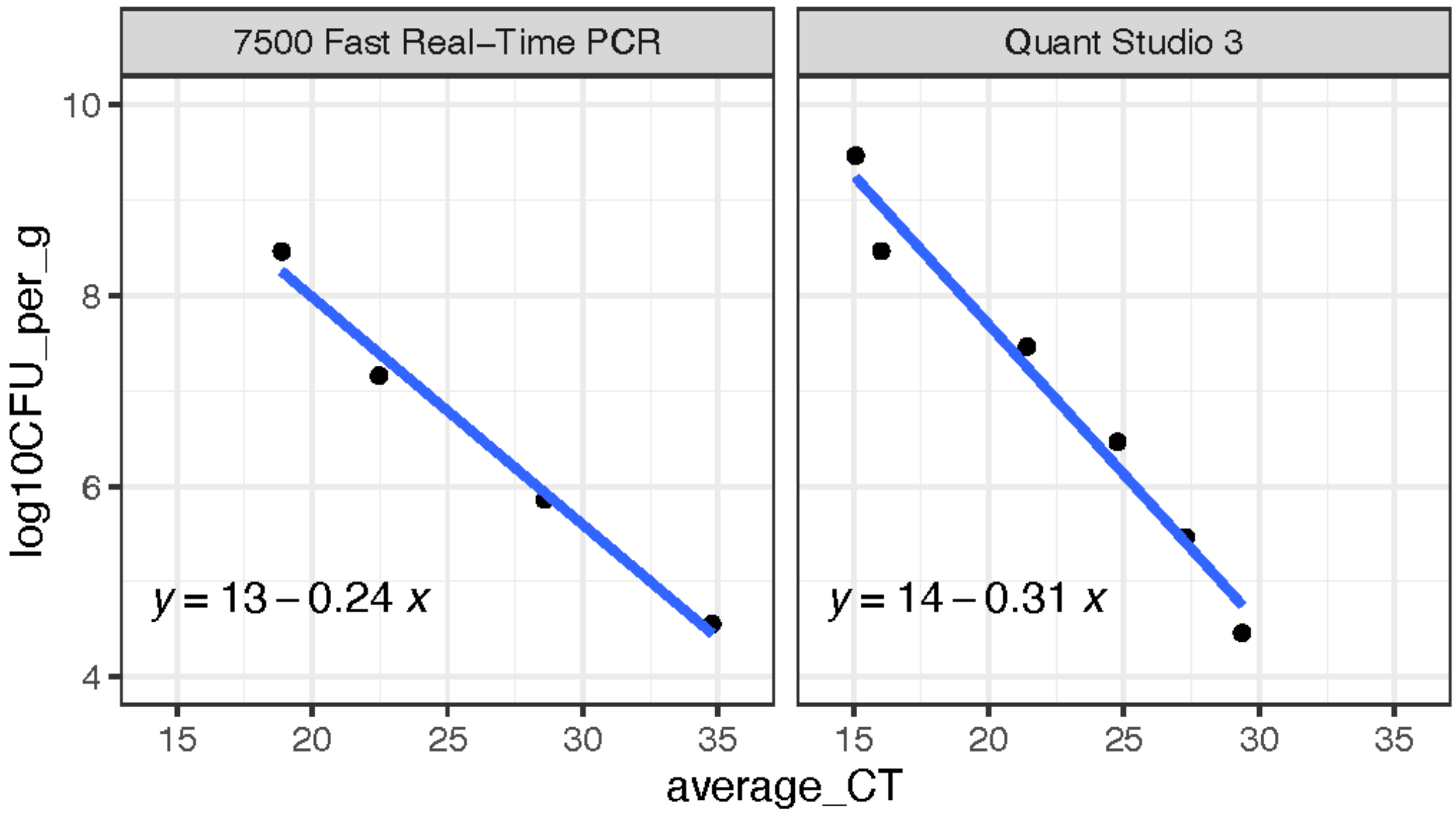

Figure S9 Correlation between Susceptibility metrics in 13 varieties

All susceptibility metrics from 2021 were aggregated by each variety (n=13). Correlations were all positive, but not statistically significant. From left to right, Pearson correlation coefficients (R) were 0.32 (p=0.29), 0.52 (p=0.07), and 0.25 (p=0.42).

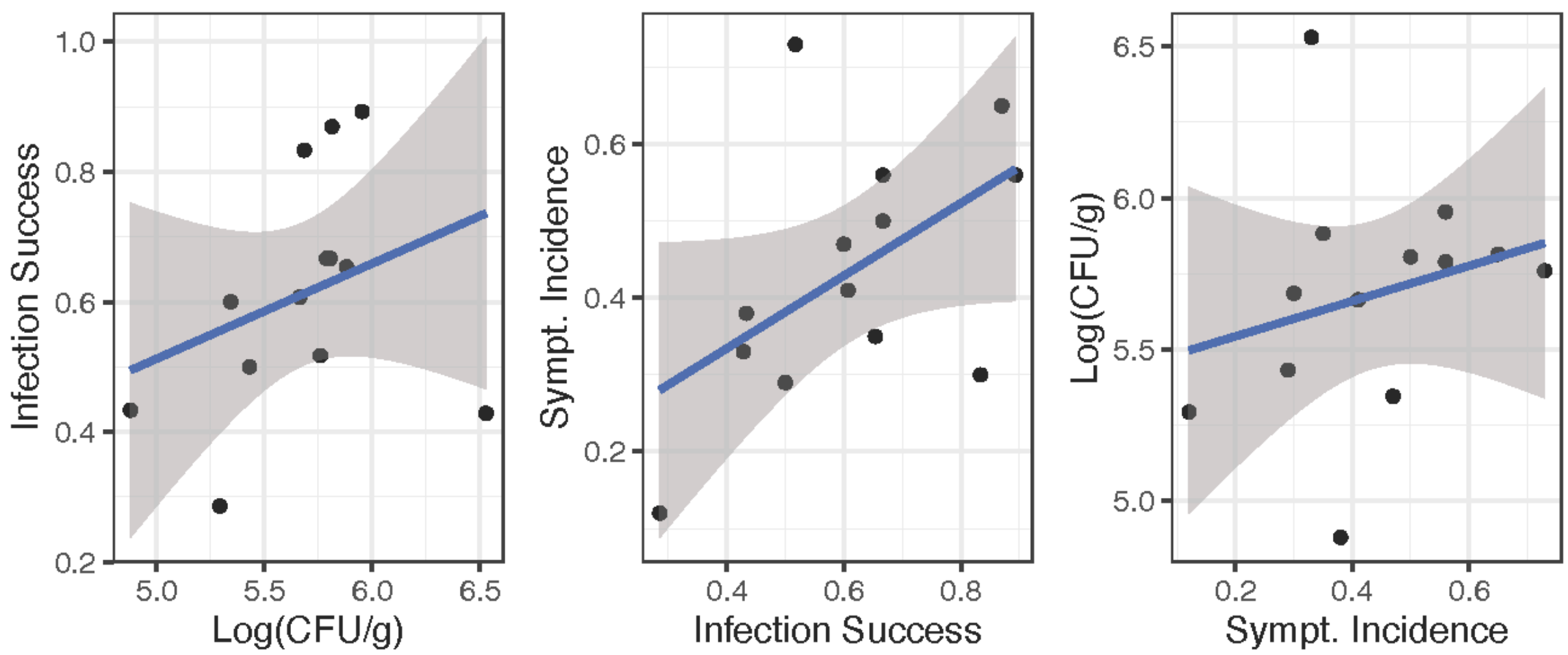

Figure S10 Distinct Pierce's disease symptoms

(A) The inoculated shoot was barren in many vines of Tinta Francisca in 2021. (B) Shrivelled berries (C) Leaf scorch (D) In 2023, we observed a lack of growth (dead positions) at the distal end of the left cordon (Western quadrant), near the point of inoculation. (E) Two infected vines in 2023 with no shoot growth on either bilateral cordon (all observed quadrants). (F/G) Early-season stunting in vines in June (F: 2023 and G: 2022) showed stunted shoots on the inoculated (left) side of the vine.

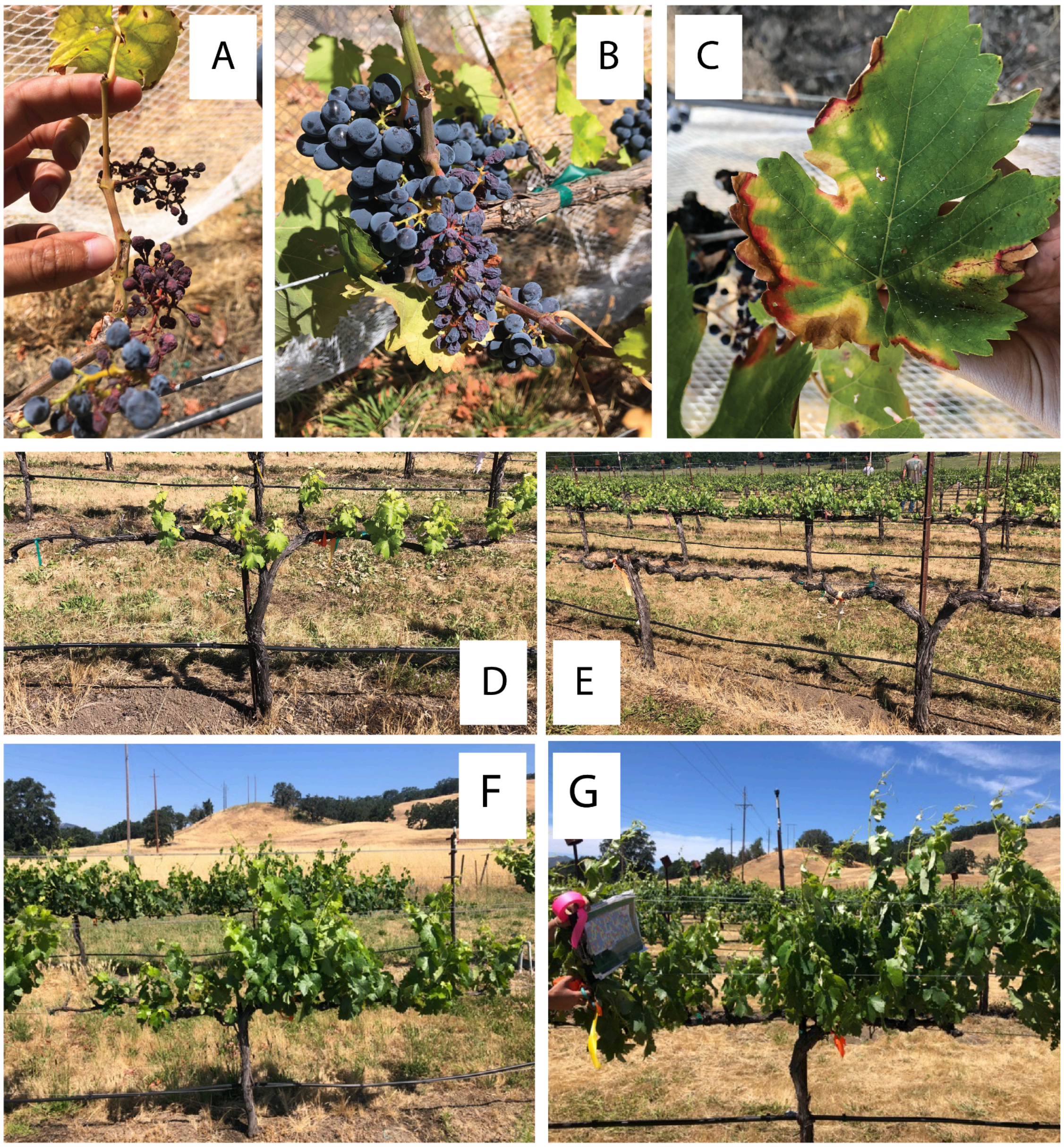

Figure S11 - Symptoms on Western Quadrant

Each symptom type is shown as a percentage of that symptom in positive vines that year. Symptom types include (1) Barren shoot, only observed in 2021 (2) “Match” for matchstick petioles (3) Scorched leaves (4) Stunting, observed visually in symptom evaluations (5) “SC” for shriveled clusters (6) Uneven maturation and (7) “dead” for no growth on that quadrant.

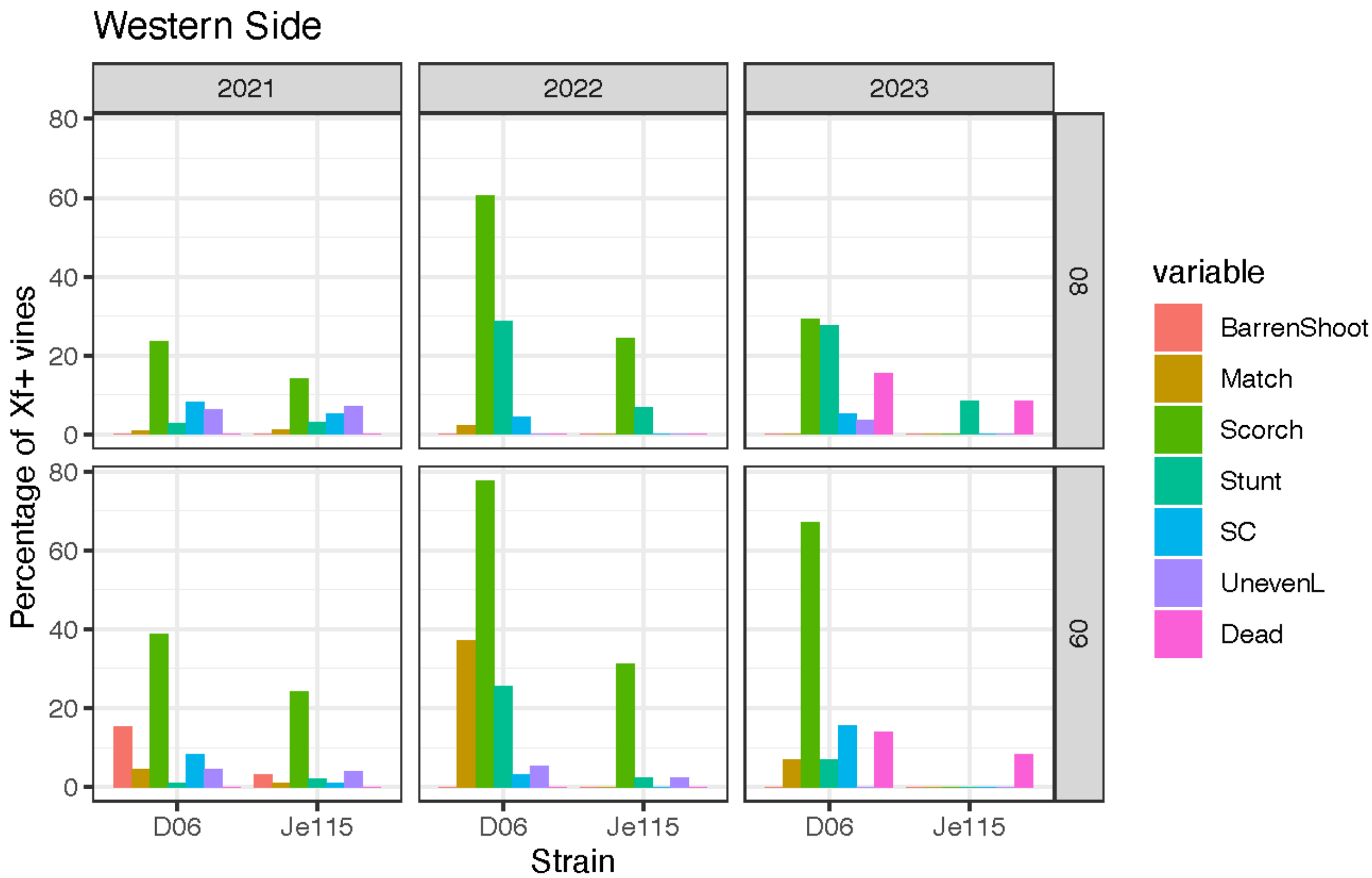

Figure S12 - Stunting measurements

Stunting was assessed by comparing shoot length in *Xf* positive vines (red) and uninfected vines (grey) in the spring of 2022 and 2023. The height for each vine was normalized to the average height in uninfected vines in that variety, calculated for each month / year combination. A normalized height of 1 means no stunting, and a normalized height below 1 indicates stunting. Across all time points, the normalized shoot height was significantly shorter (by ~17% in 2022 and by ~40% in 2023) in infected compared to uninfected vines (GLMM: Wald X^2=13.1615, df=1, p=0.0003). Early season stunting was assessed visually in Kahn et al. 2023, but had not previously been recognized as a PD symptom.

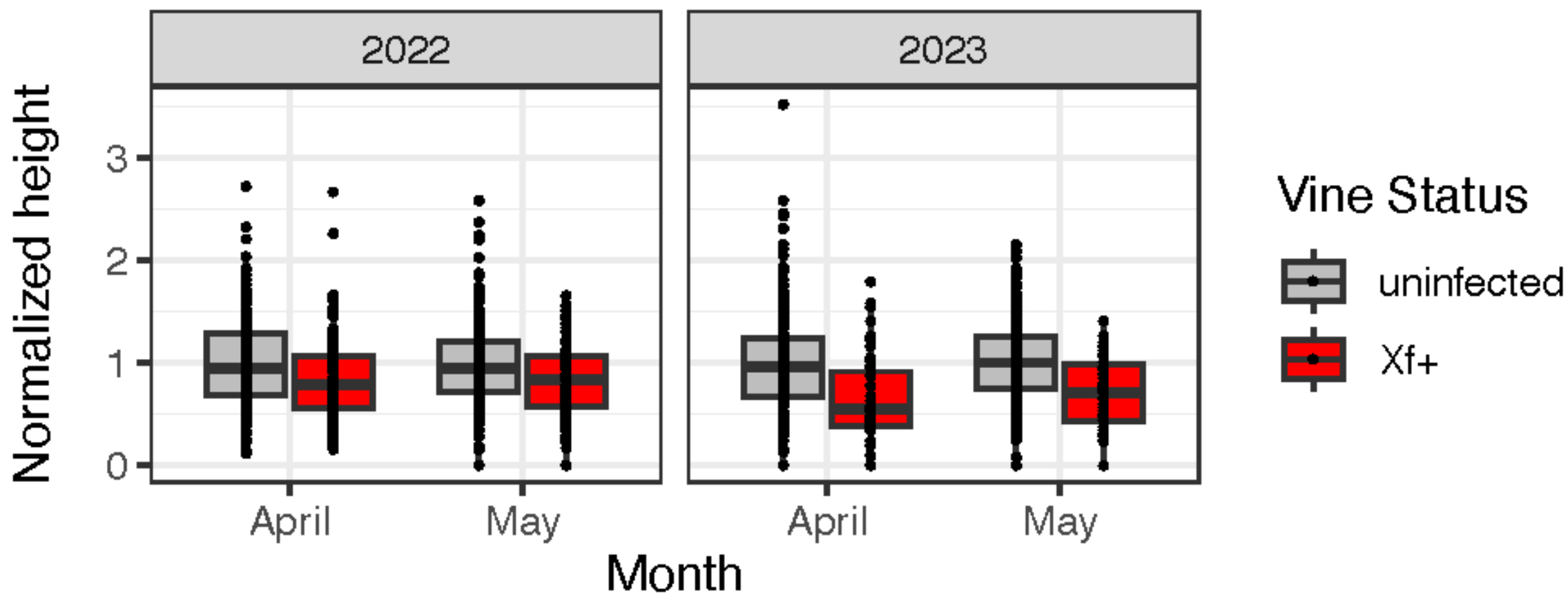

Figure S13 Positive Vine Counts

Number of positive vines plotted (A) by year (B) by quadrant of the vine and year in Albariño, Tannat, and Tinta Francisca ("TF") (C) by year and variety. For all plots, the purple bars represent D06-infected vines, and the red bars represent Je115-infected vines.

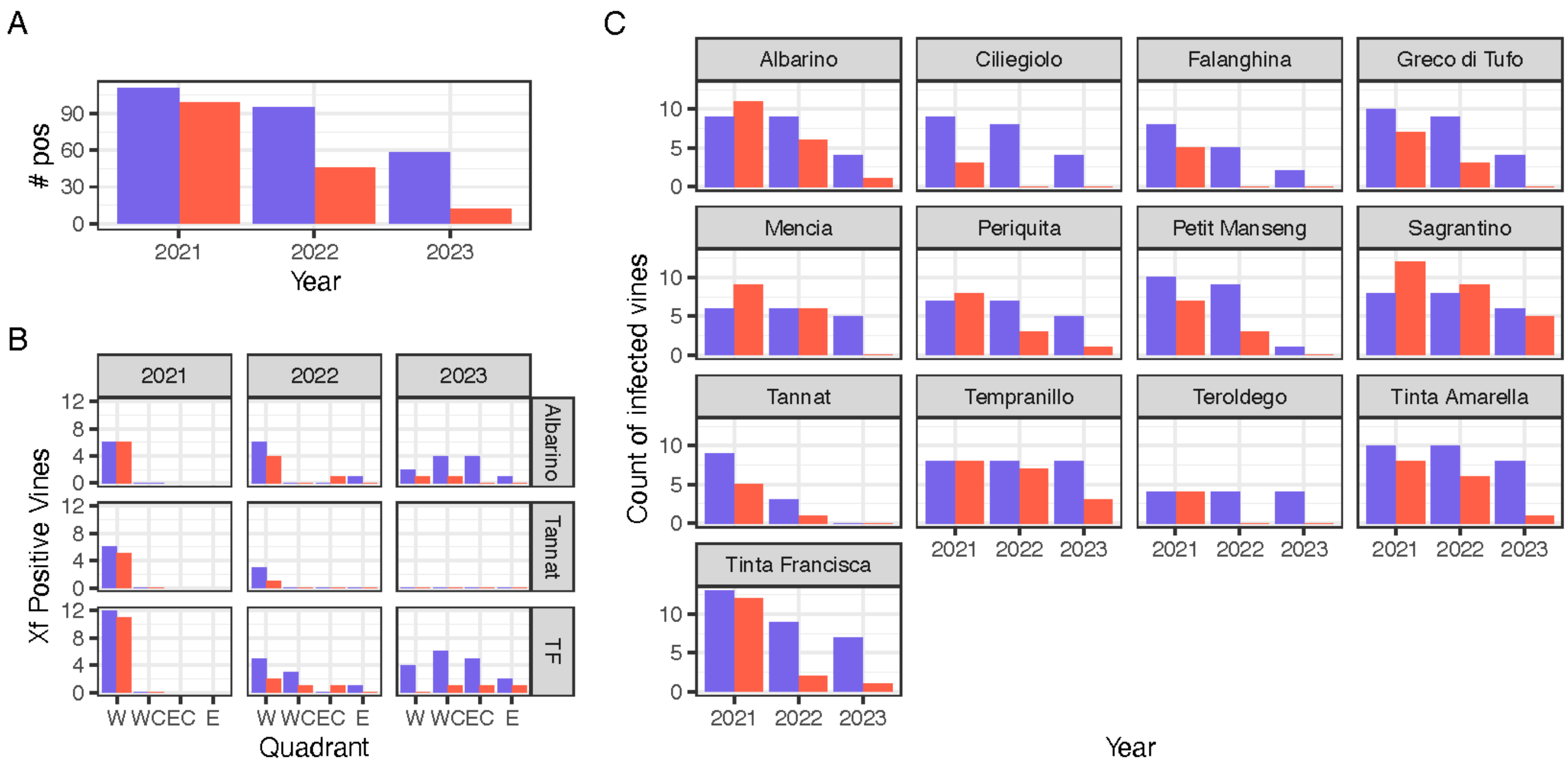

Figure S14 Correlation between Susceptibility and Recovery

All three susceptibility metrics from 2021 were negatively correlated with overall recovery, aggregated by each variety (n=13). Correlations were all positive, but not statistically significant. From left to right, Pearson correlation coefficients (R) were 0.14 (p=0.65), 0.21(p=0.49), and 0.41 (p=0.16).

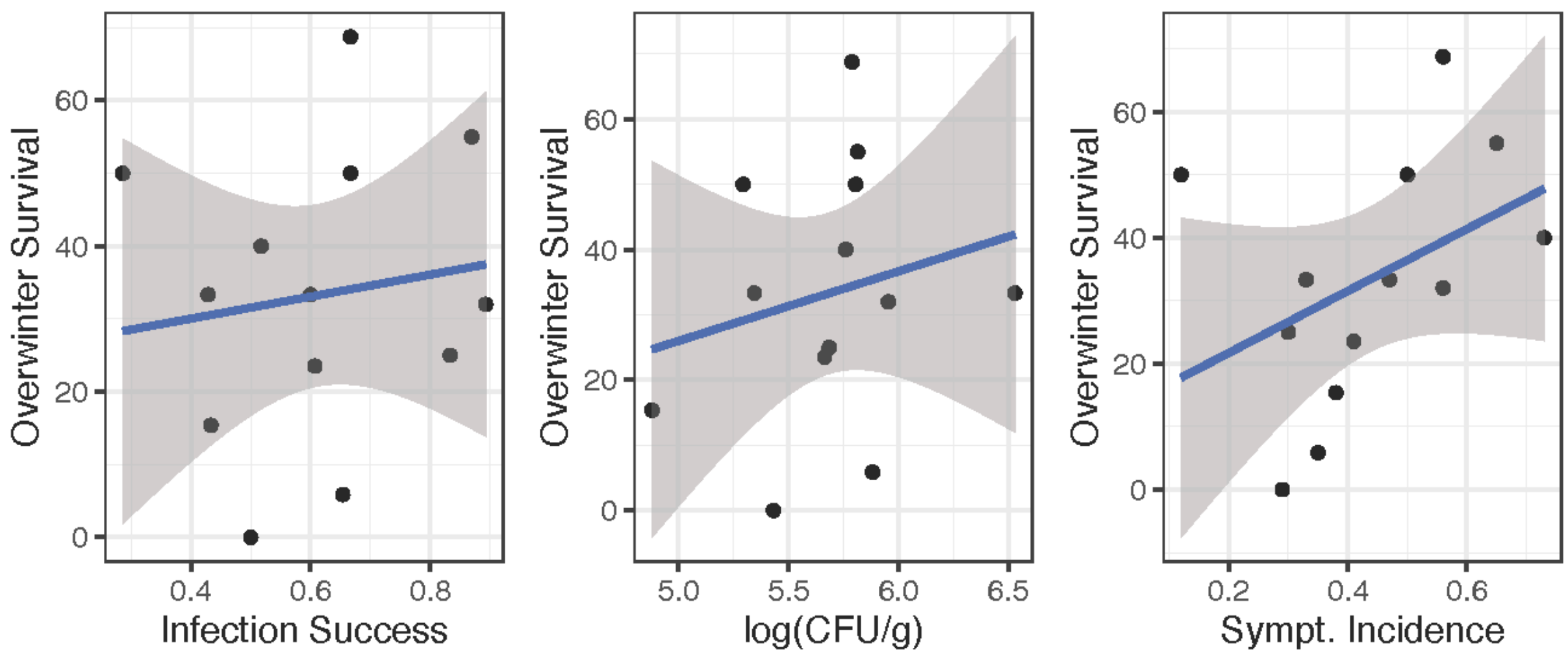

Figure S15 Greenhouse Experiment #1 with Derived Isolates

This experiment used cv. Ciliegiolo plants. The two panels split the derived isolates by parent strain (D06 or Je115); unfortunately, none of the Je115 wildtype-treated plants were positive (0/5) for comparison.

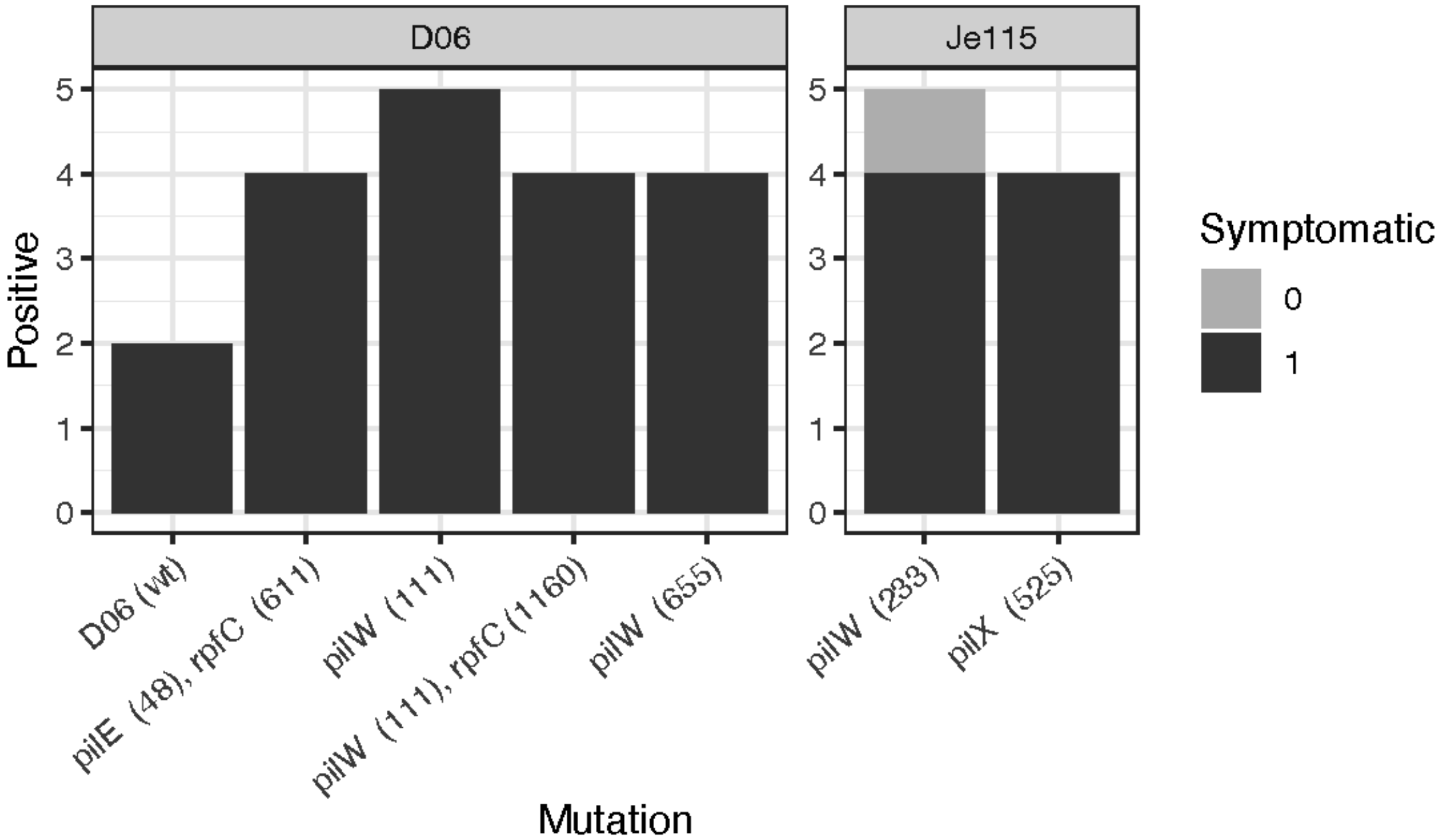

Figure S16 Temperature data from Hopland and Bakersfield

Temperatures were logged hourly in both locations in the winter months (October through April) from 2022 through 2025. Freezing (below 0°C) and chill (< 7.22°C) hours were measured across both sites; both metrics show that Hopland experiences colder winter temperatures than Bakersfield.

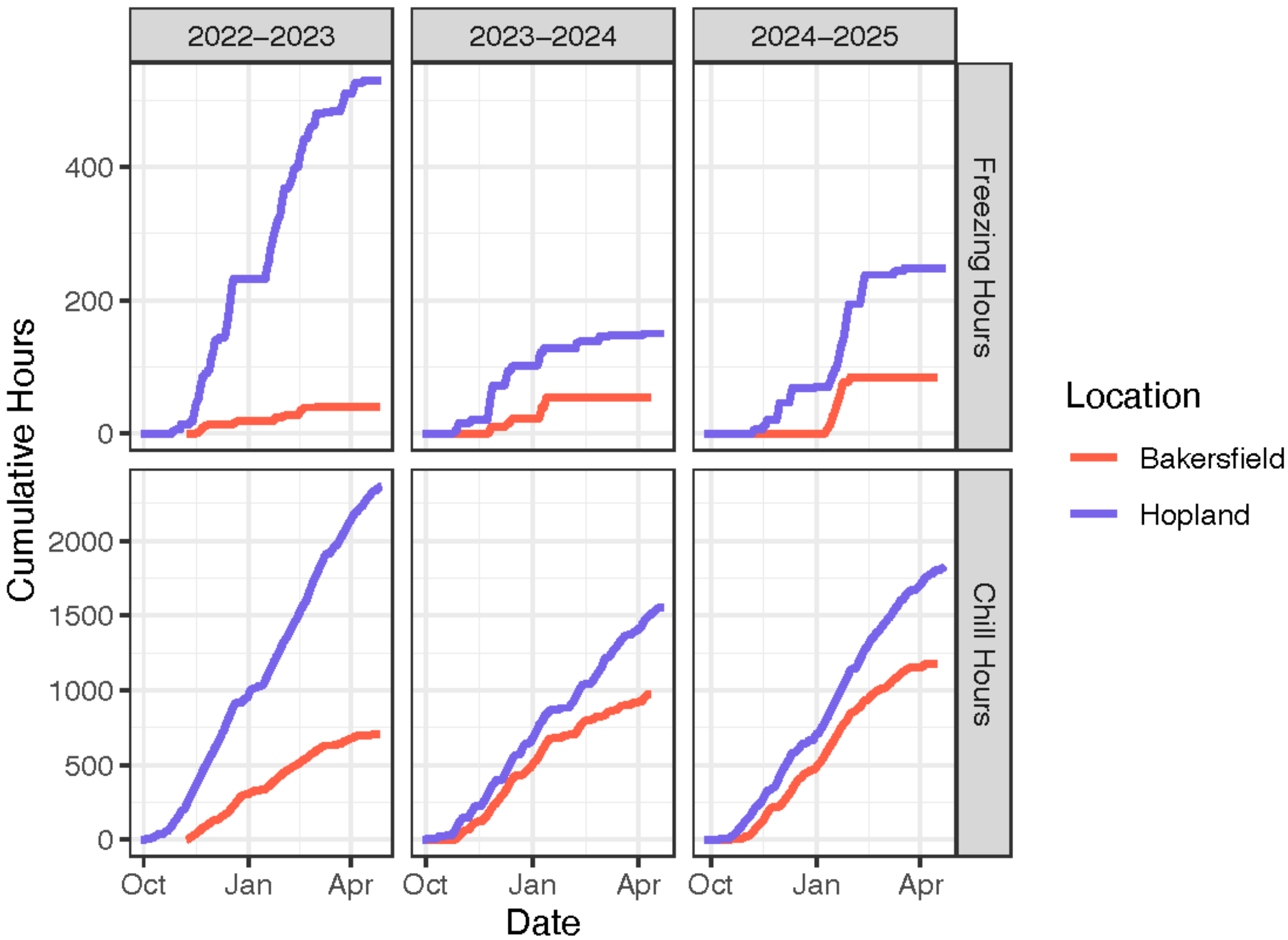

Supplement: Supplementary file 1 [file aem.01520-26-s0001.pdf]
